# Design and Implementation of a Low-Cost Priapism Reduction Task Trainer

**DOI:** 10.21980/J8K64F

**Published:** 2021-01-15

**Authors:** Andrew Eyre, Valerie Dobiesz

**Affiliations:** * Brigham and Women’s Hospital/Harvard Medical School, Department of Emergency Medicine, Boston, MA; ^ STRATUS Center for Medical Simulation/Brigham and Women’s Hospital, Department of Emergency Medicine, Boston, MA

## Abstract

**Audience:**

This low-cost priapism reduction task trainer is designed to instruct emergency medicine (EM) resident physicians.

**Introduction:**

Priapism is a true urologic emergency that EM physicians must be able to diagnose and treat in order to prevent significant tissue damage and loss of erectile function. Given the nature of the condition, priapism treatment is often both physically invasive and psychologically upsetting. Simulation allows learners to practice invasive or rare procedures in a safe and educational environment. At present, there are few inexpensive and easily created task trainers for priapism reduction. Our goal was to create an inexpensive, reusable task trainer that allows learners to practice the skills needed for priapism reduction.

**Educational Objectives:**

By the end of this educational session, learners should be able to 1) Verbalize the difference between low-flow and high-flow priapism 2) Describe the landmarks for a penile ring block and cavernosal aspiration/injection 3) Demonstrate the appropriate technique for performing a penile ring block, cavernosal aspiration, and cavernosal injection.

**Educational Methods:**

Using inexpensive and commonly found materials, we were able to successfully create a partial task trainer for teaching priapism reduction techniques including administering local anesthesia, medication injection, and realistic cavernosal aspiration with simulated blood return. As part of a standard EM residency didactics curriculum, this task trainer has been used to teach post graduate year (PGY) 1–4 resident learners. After an introductory didactic session, participants were given the opportunity for hands-on skills-based practice using the simulated task trainer.

**Research Methods:**

Learners were asked to complete a post-session survey to assess the educational value of the station and the task trainer.

**Results:**

We were able to successfully create a low cost, easy to build partial task trainer for priapism reduction that allowed learners to perform local anesthesia, medication injections, and corporal aspiration. Twenty-five residents (ten PGY-1, five PGY-2, five PGY-3, five PGY-4) participated in a single didactic session and completed a post-session survey. The majority (68%, N=17) of participants had never previously treated a patient with priapism. On average, participants rated their comfort managing a patient with priapism before the session to be 1.76 on a 5-point Likert-scale (where 1=not at all comfortable and 5=extremely comfortable). Following the session, participants’ comfort increased to 3.76 on the same scale. Participants rated the usefulness of the priapism model for teaching priapism reduction techniques to be 4.64 on a 5- point scale (where 1=not at all useful and 5=extremely useful).

**Discussion:**

Using inexpensive and commonly found materials, we were able to successfully create a partial task trainer for teaching priapism reduction techniques including local anesthesia, medication injection, and cavernosal aspiration. Learners reported that the educational session greatly increased their confidence in caring for patients presenting with priapism. Additionally, they found the priapism model to be extremely helpful for teaching reduction techniques. Our model was successful in teaching a procedure that providers may encounter in clinical practice yet most resident learners had not yet had the opportunity to perform in training.

**Topics:**

Penile anesthesia, priapism reduction, urologic emergencies.

## USER GUIDE

**Table:** 

List of Resources:	
Abstract	1
User Guide	3
Instructor Guide	9

**Learner Audience:**

Interns, Junior Residents, Senior Residents

**Time Required for Implementation:**

The task trainer can be assembled in approximately 30 minutes, although additional drying time is necessary. Once dried, preparation and setup for the station requires an additional 5–10 minutes. It is recommended that the educational session be scheduled for 45 minutes; however, this can be adapted to the level of learners and time constraints. Given that most EM resident learners have limited opportunity to regularly perform this procedure in the clinical setting, we recommend including this educational session a minimum of annually or bi-annually as part of a standardized didactic curriculum.

**Recommended Number of Learners per Instructor:**

We recommend a ratio of 1 task trainer for every 3 or 4 learners with a maximum instructor to learner ratio of 1:9.

**Topics:**

Penile anesthesia, priapism reduction, urologic emergencies.

**Objectives:**

By the end of the educational session, learners should be able to:

1. Verbalize the difference between low-flow and high-flow priapism
2. Describe the landmarks for a penile ring block and cavernosal aspiration/injection
3. Demonstrate the appropriate technique for performing a penile ring block, cavernosal aspiration, and cavernosal injection.

### Linked objectives and methods

This educational session pairs a brief introductory lesson with an opportunity for hands-on practice using a simulated priapism partial task trainer. The Priapism Study Guide reviews the differences between high and low flow priapism, anatomy and technique for performing a penile ring block, and the anatomy and technique for performing penile aspirations and injections (objectives 1 and 2). The subsequent practice using the partial task trainer allows learners to apply and demonstrate their knowledge (objective 3).

Simulation-based medical education is a widely accepted and effective teaching modality for both cognitive and procedural skills. Built upon a wide array of educational theories and literature, simulation is an active teaching strategy that enhances learning by providing a contextual framework and allows learners to process and use knowledge in an engaging way. As described by Bloom’s Taxonomy, learners acquire and process information at differing levels. Lectures primarily transmit information and ask learners to remember or recall new information while simulation-based medical education allows learners to demonstrate not only the acquisition but also the application of knowledge, both of which represent higher levels of understanding on Bloom’s Taxonomy.

Simulation provides learners with a consistent and safe environment for practice and learning. Whereas the clinical environment is highly variable and instructors cannot guarantee that learners will encounter certain pathology or cases, simulation-based medical education can ensure that all learners are exposed to the necessary experiences. Additionally, simulation provides learners the opportunity to practice skills without introducing additional risks to their patients or themselves.

While simulation can be used to teach a wide variety of skills and concepts, it is the ideal modality for teaching low frequency or high acuity procedures. Priapism reduction is a challenging procedure to teach at the bedside due to the infrequent and time-sensitive nature of treatment as well as the physical and emotional distress experienced by these patients. By teaching priapism reduction techniques using simulation, we can ensure that our learners demonstrate a basic level of understanding and skill before performing them on actual patients.

### Recommended pre-reading for instructor

- Priapism Study Guide (Powerpoint)
- UpToDate Article on Priapism
- http://www.emdocs.net/priapism-ed-pearls-pitfalls/

### Learner responsible content (LRC)

- Priapism Study Guide (Powerpoint)
- UpToDate Article on Priapism
- http://www.emdocs.net/priapism-ed-pearls-pitfalls/

### Implementation Methods

Prior to the educational session, the task trainers need to be created and prepared with simulated blood for aspiration. Guided by institutional availability and practices, the equipment necessary to perform the penile injection and aspiration should be assembled and laid out for each station. Assuming each session lasts 45 minutes, we recommend the instructor spend 15 minutes reviewing the common etiologies of priapism, the relevant anatomical structures, and the critical steps for performing a penile block, penile aspiration, and penile injection. As part of this didactic, we recommend the instructor demonstrate each of these procedures on the task trainer. Following the introduction, learners should practice each of these procedures on the priapism task trainer, with the instructor circulating between groups to answer questions and provide feedback. At the conclusion of the education session, the instructor should provide a recap of the high-yield learning points, as guided by the learning objectives and allow time for any remaining questions.

### List of items required to replicate this innovation

Many of the recommended materials are commonly available without the need for specific purchase or can be easily substituted for equivalent items.

1. Household sponge (https://www.cvs.com/shop/total-home-kitchen-sponges-prodid-155662)
2. 1-inch Kling gauze (https://www.buyemp.com/product/conform-stretch-bandages)
3. Endotracheal tube stylet (https://www.buyemp.com/product/curaplex-stylettes)
4. Sterile ultrasound probe cover (https://www.edmus.com/product/pull-up-ultrasound-probe-cover/)
5. 5-inch segment of simulated bowel (https://sim-vivo.com/simb.html)
6. “Liquid-Latex” paint (https://www.walmart.com/ip/Liquid-Latex-Makeup-Perfect-for-Halloween-Great-for-Zombie-Skin-Scarring/794964920)
7. Glue (https://www.cvs.com/shop/gorilla-super-glue-15g-prodid-1920021)
8. Sterile blue basin (https://www.amazon.com/Medline-DYND50321-Sterile-Plastic-Large/dp/B00TPHUTKK)
9. Foam arm padding (https://www.universalmedicalinc.com/disposable-ulnar-nerve-elbow-foam-protector-6-x-15-5-x-2-thick.html)
10. Plastic zip ties (https://buycableties.com/products/6-inch-tear-away-cable-ties-colorblue?variant=6185279108&currency=USD&utm_medium=product_sync&utm_source=google&utm_content=sag_organic&utm_campaign=sag_organic&utm_term=4577816664294263)
11. Medical tape (https://www.buyemp.com/product/3mdurapore-tape)
12. Simulated blood (https://vatainc.com/product/simulated-blood-stain-resistant-one-quart/)
13. 10cc syringe (https://www.buyemp.com/product/10cc-bd-luer-lok-disposable-syringe)

**Figure:**
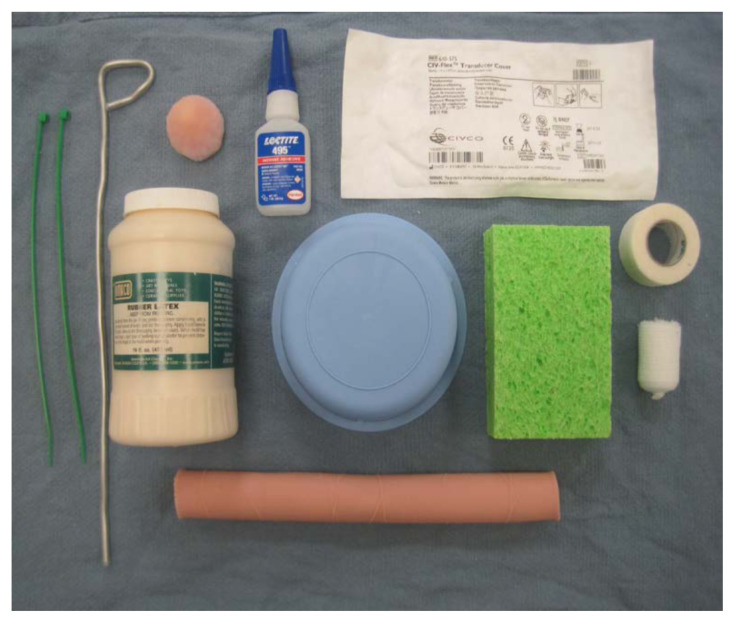

### Approximate cost of items to create this innovation

This task trainer is created using materials that are commonly available or bought in bulk and can be assembled for under $130 (US dollars), depending on local purchasing agreements and existing simulation resources.

### Detailed methods to construct this innovation

1. Roll the sponge tightly length-wise
  **Figure:**
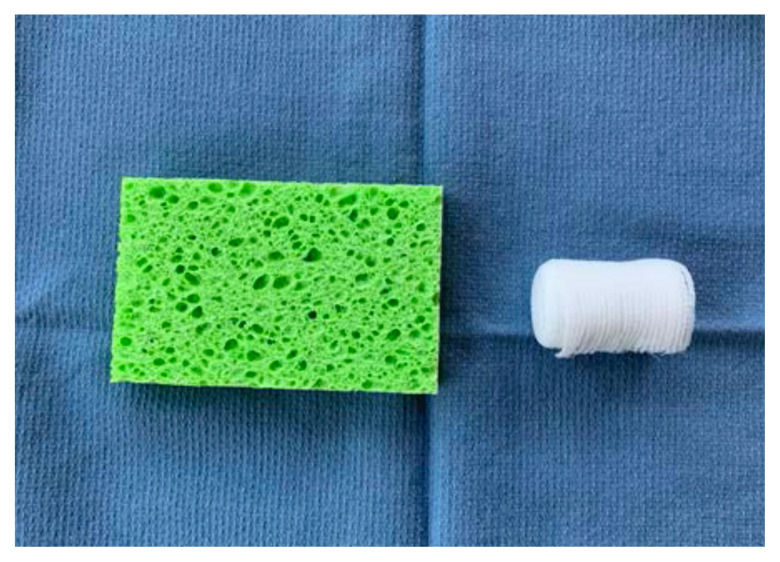
2. Wrap the sponge roll with the 1-inch bandage
  **Figure:**
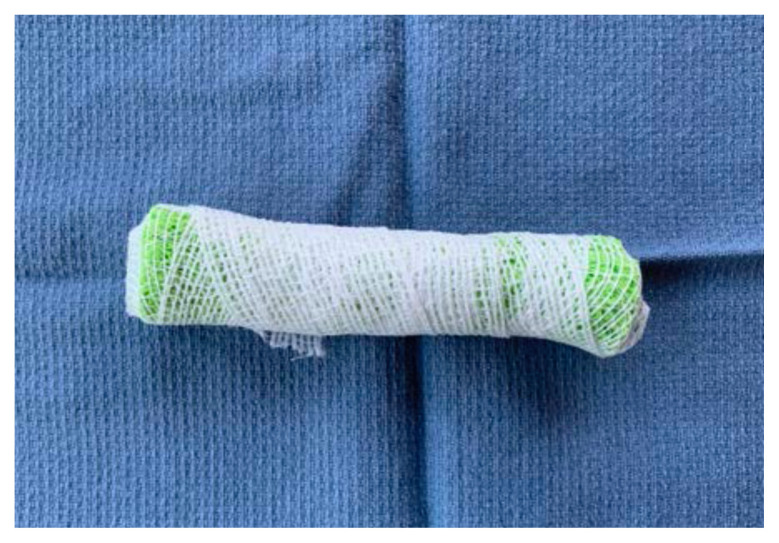
3. Place the sponge roll inside the ultrasound probe cover, cutting the cover to leave 1 inch of extra plastic. Tie the open end in a knot
  **Figure:**
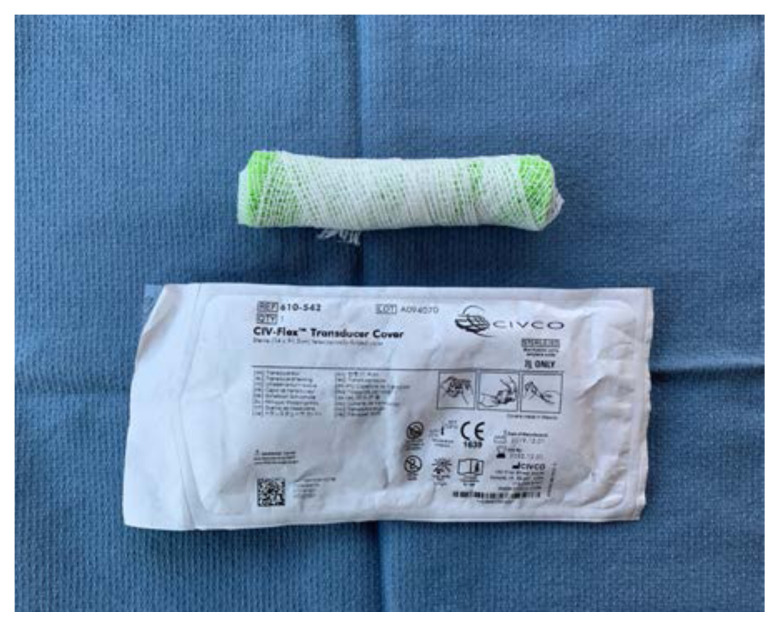
  **Figure:**
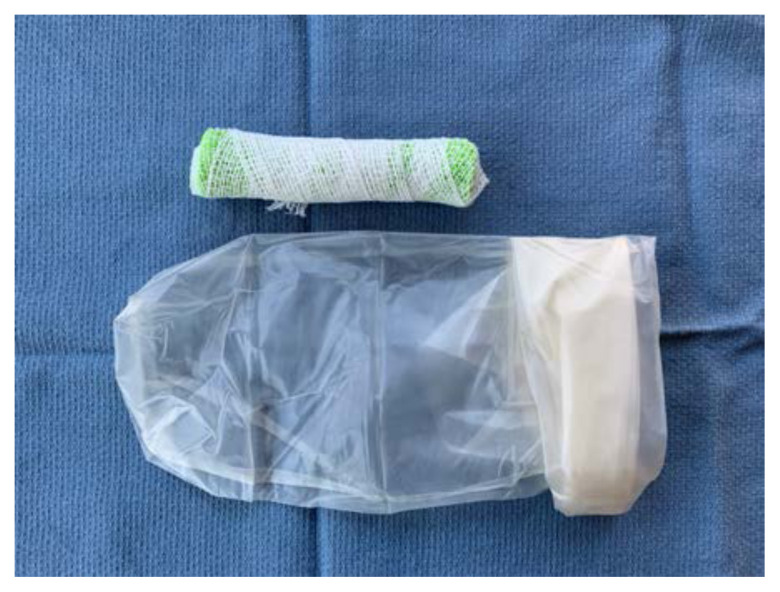
4. Using tape, attach the stylet along the bottom-side of the sponge roll with the extra stylet extending off to one side
  **Figure:**
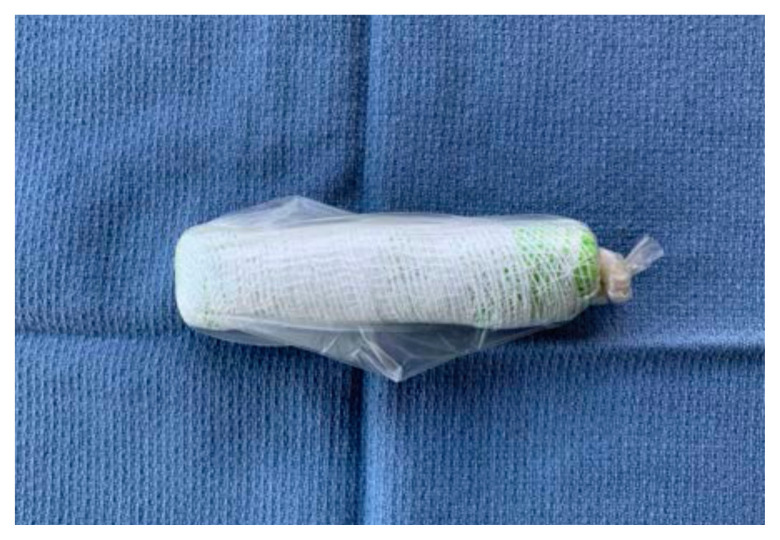
5. Place the length of simulated bowel over the sponge roll to simulate skin. If desired, leave ½ inch of simulated bowel extending off the distal end. This can be rolled backwards and glued down to simulate foreskin
  **Figure:**
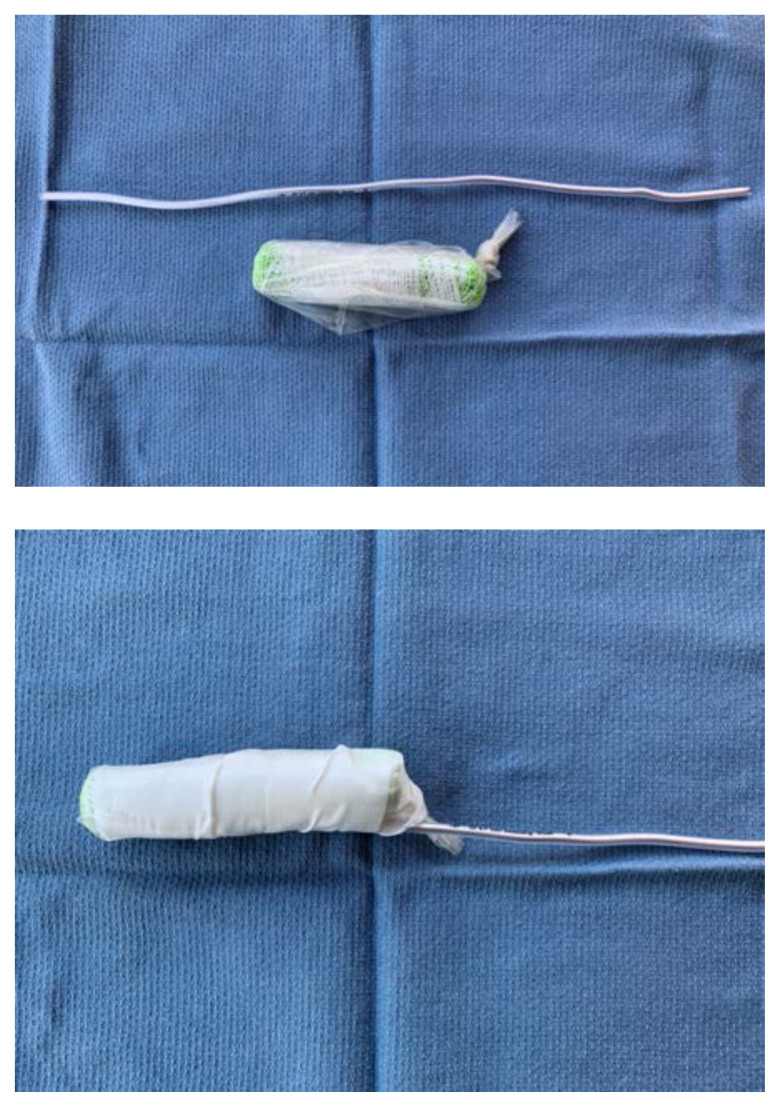
6. To simulate the glans penis, cut a pyramidal shaped piece off of the foam arm pad that matches the circumference of the sponge roll. Dip this piece of foam into the latex paint, using enough coats to fully cover it
  **Figure:**
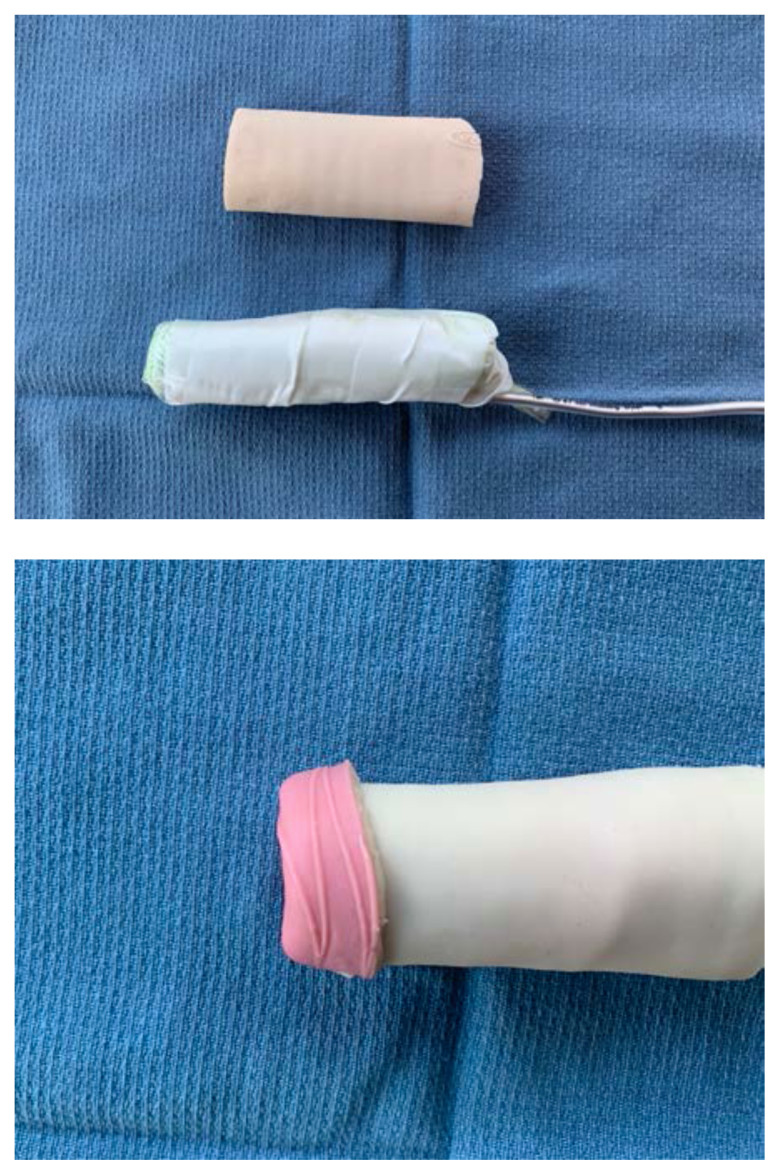
7. Once dry, attach the foam piece to the distal end of the sponge roll using glue.
8. Apply additional glue or latex paint to the base of the sponge roll to create a water-tight seal.
9. To attach the model to the sterile basin, poke a hole in the top of the basin and two small holes ½ cm apart on the side. Place the long end of the stylet through the top hole and then use a zip tie or glue to secure the end of the stylet to the side of the basin through the small holes
  **Figure:**
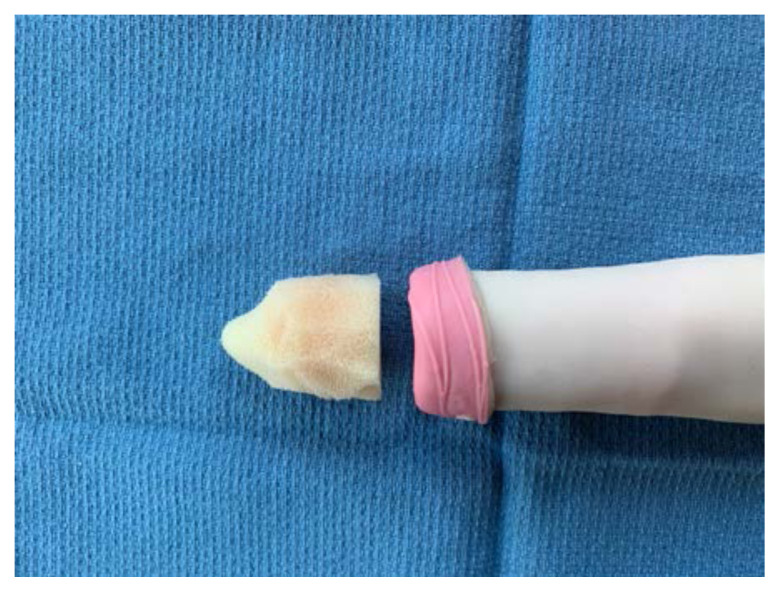
  **Figure:**
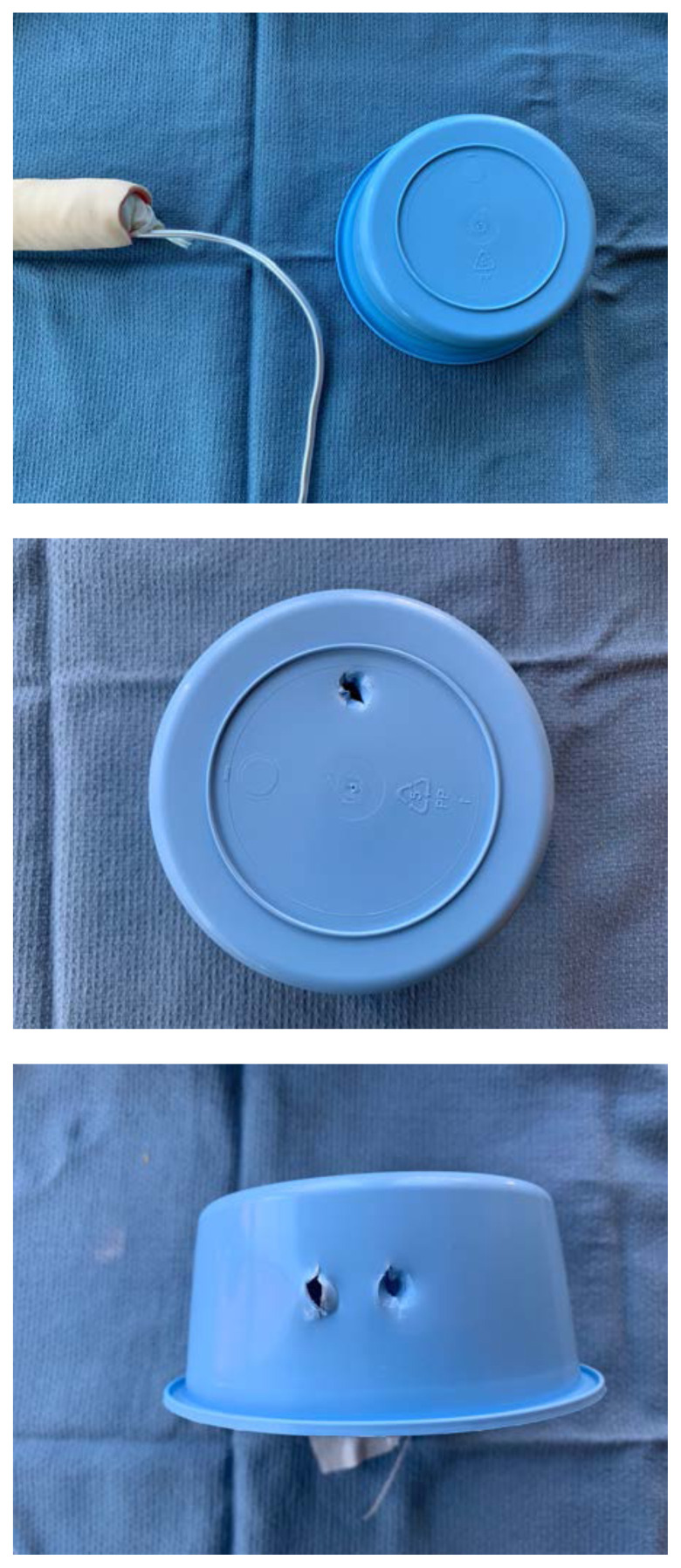
10. To prepare the model for use, mix simulated blood with water and inject the fluid into the sponge using a syringe and needle. Additional fluid may need to be injected between users
  **Figure:**
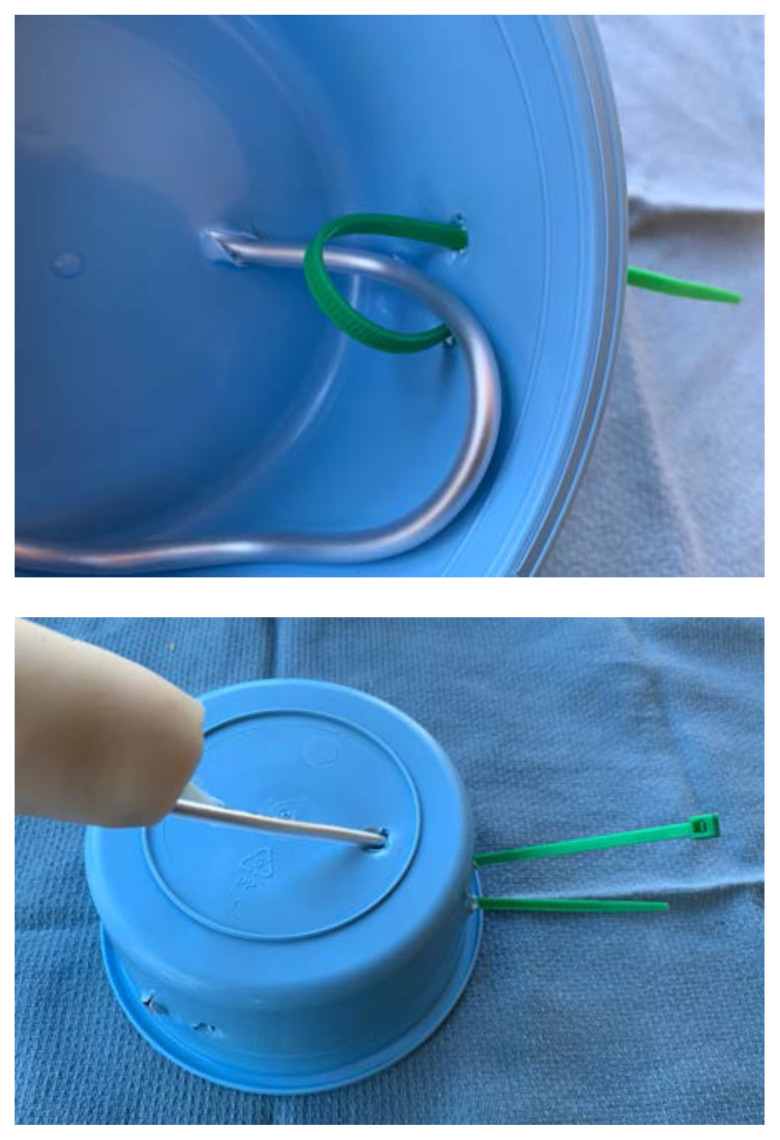
  **Figure:**
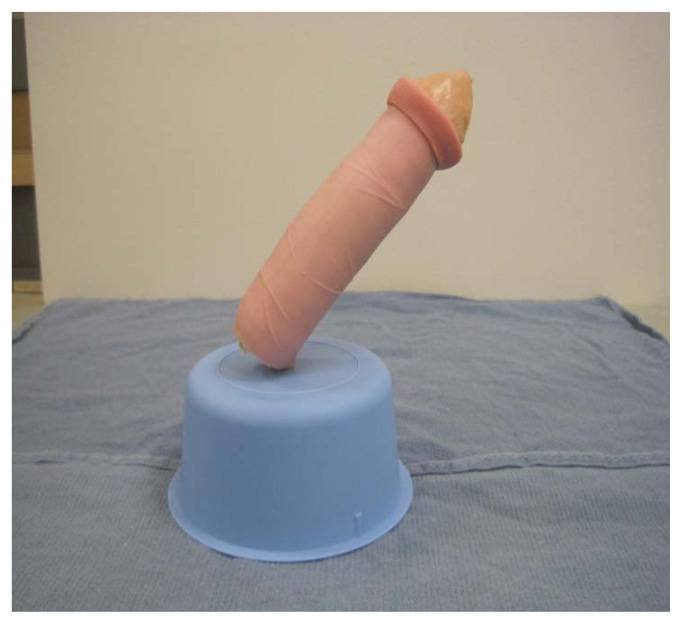

**Figure:**
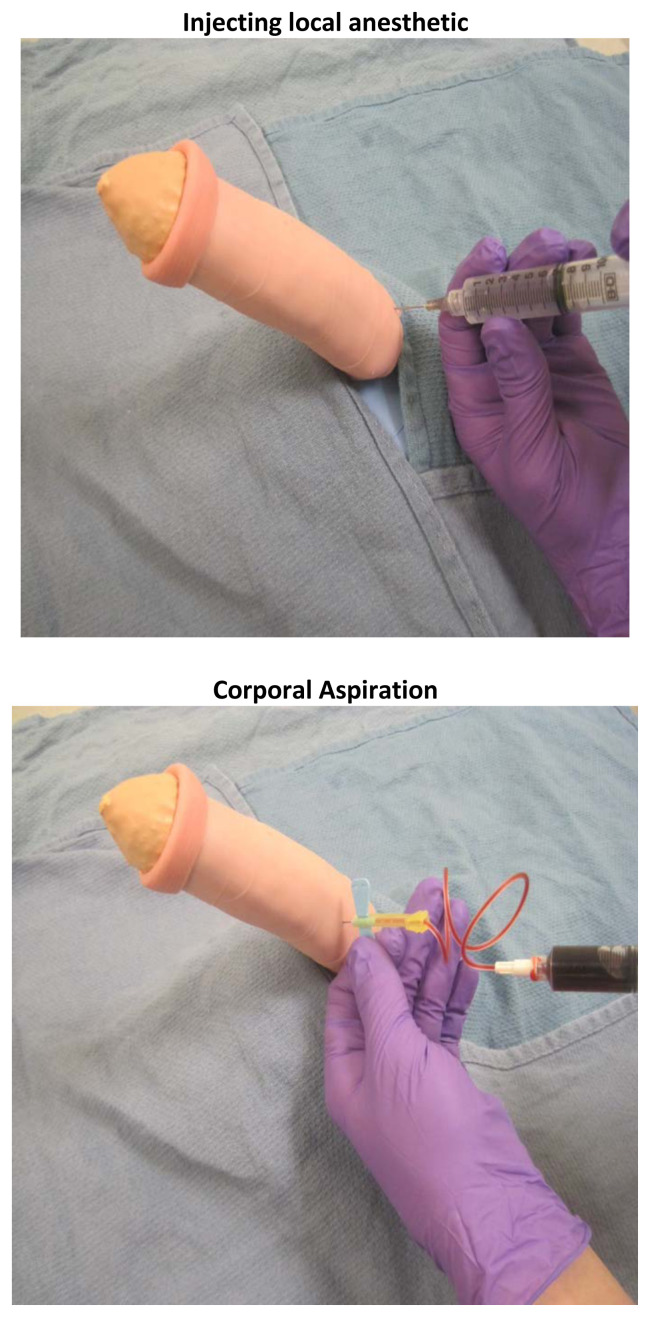

### Associated content

- Priapism Study Guide (Powerpoint)
- https://youtu.be/73c8-GwE6tY
- https://youtu.be/3p0qEfISggs

### Results and tips for successful implementation

This partial task trainer has been used for the past 3 years as part of a simulation-based didactics program for PGY 1–4 EM residents. Following the initial session, learners were asked to complete a post-session survey. The survey contained basic demographic and experience questions, Likert-type questions, and an open-response question. Twenty-five residents (10 PGY-1, 5 PGY-2, 5 PGY-3, 5 PGY-4) participated in the initial didactic session and completed the post-session survey. The majority of learners (68%, N=17) had never previously treated a patient with priapism. On average, learners rated their comfort managing a patient presenting with priapism before the session to be 1.76 on a 5-point Likert-scale (where 1=not at all comfortable and 5=extremely comfortable). Following the session, learners’ comfort increased to 3.76 on the same scale. Learners rated the usefulness of the priapism model for teaching priapism reduction techniques to be a 4.64 on a 5- point scale (where 1=not at all useful and 5=extremely useful). In the open-response section, learners provided positive feedback for the station and task trainer. One learner recommended adding a simulated scrotum to the task trainer to assist with landmarks and orientation.

### Discussion

Using inexpensive and commonly found materials, we were able to successfully create a partial task trainer for teaching priapism reduction techniques including local anesthesia, medication injection, and cavernosal aspiration. Learners reported that the educational session greatly increased their confidence in caring for patients presenting with priapism. Additionally, they found the priapism model to be extremely helpful for teaching reduction techniques. Our model was successful in teaching a procedure that providers may encounter in clinical practice yet most resident learners had not yet had the opportunity to perform in training.

## INSTRUCTOR GUIDE

### Evaluation and Management of Priapism in the Emergency Department

Please see associated lecture
