# Supplementary material for: Design and Implementation of a Low-Cost Priapism Reduction Task Trainer
Source: J Educ Teach Emerg Med. 2021 Jan 15;6(1):I1–9. doi: 10.21980/J8K64F (PMC10332760; doi:10.21980/J8K64F)
Supplement: Supplementary file 1 — Please see associated lecture [file jetem-6-1-i1-supp1.pptx]

## Slide 1
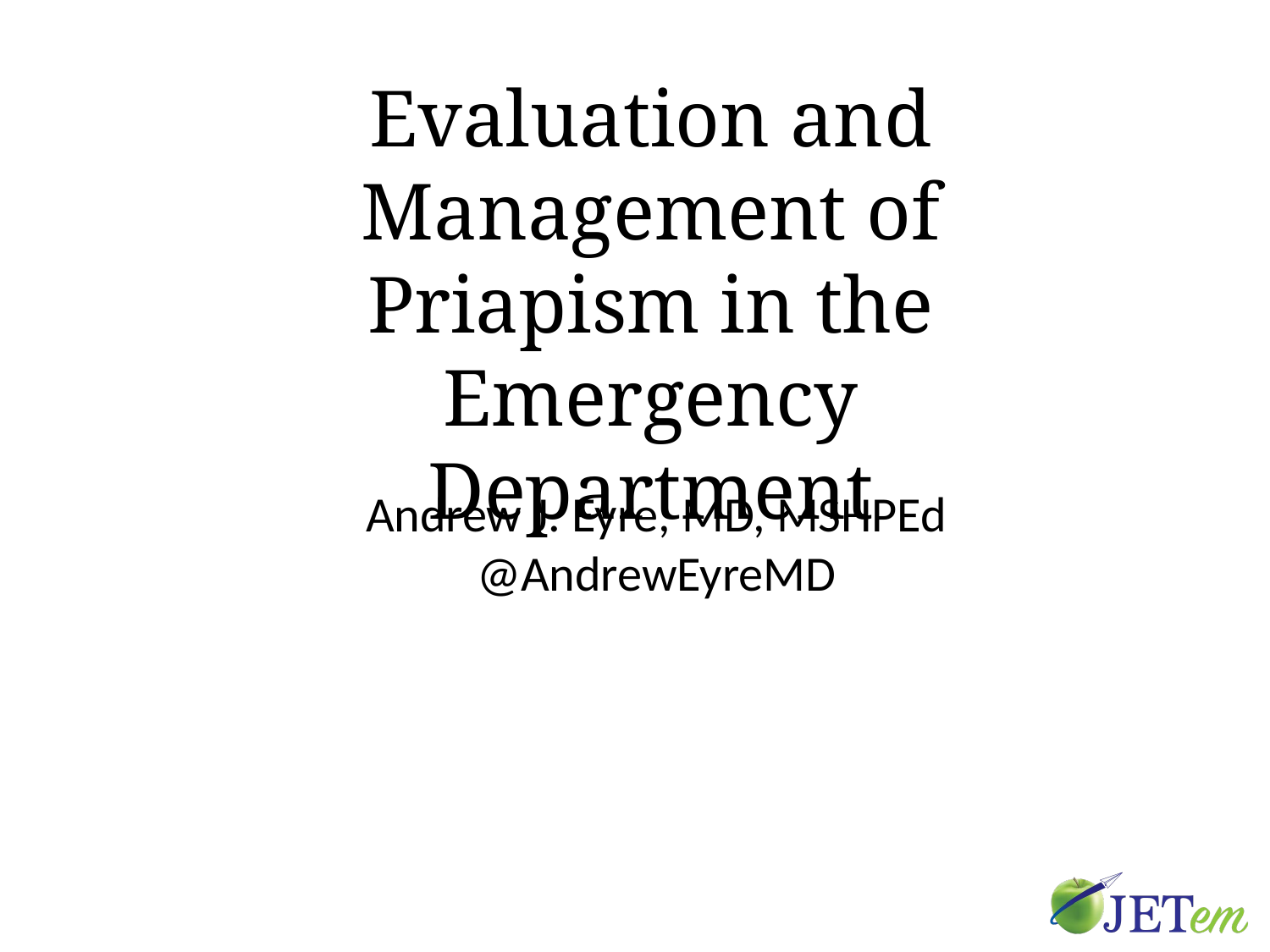

Evaluation and Management of Priapism in the Emergency Department
#
Andrew J. Eyre, MD, MSHPEd
@AndrewEyreMD

## Slide 2
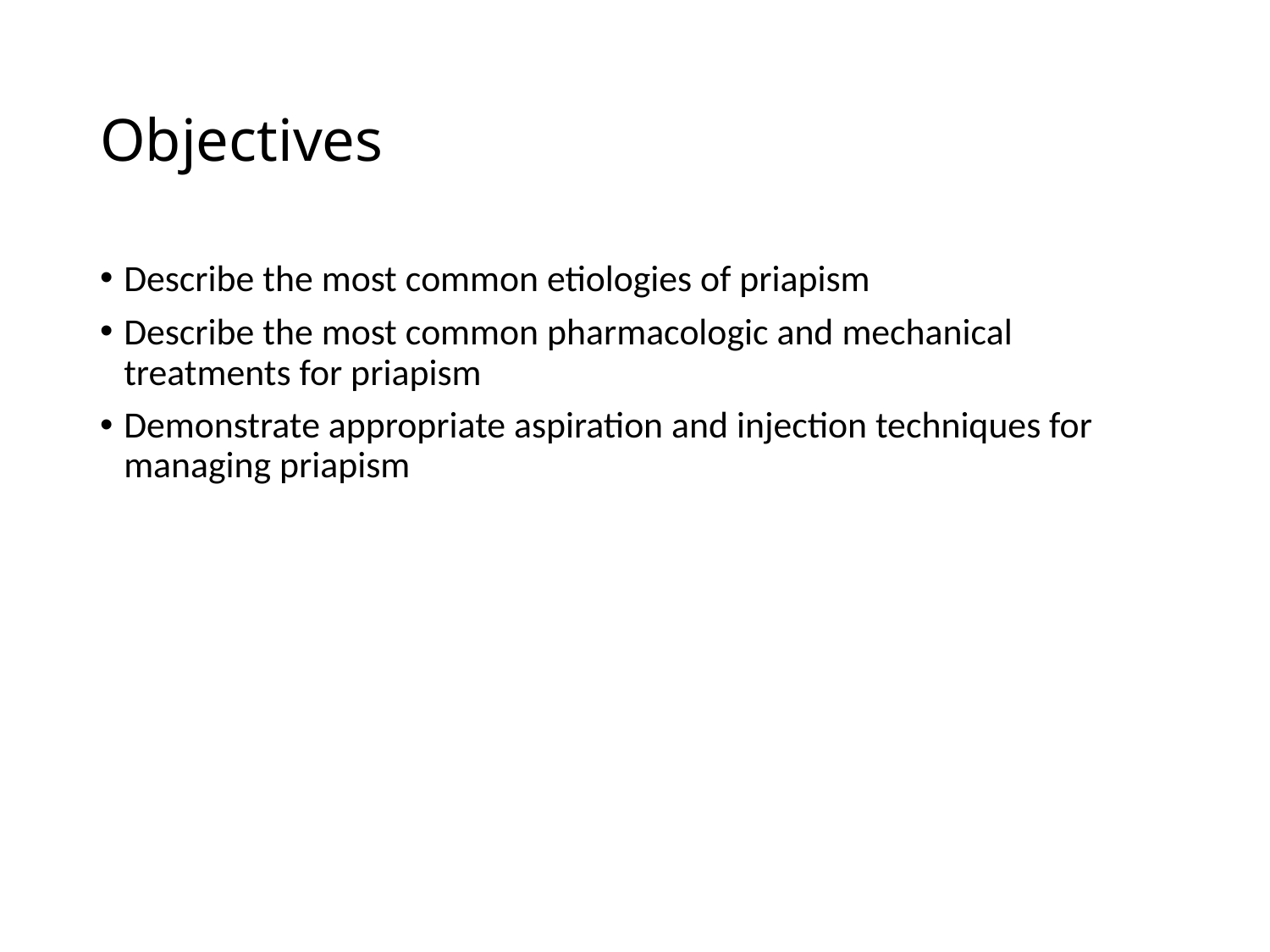

# Objectives
Describe the most common etiologies of priapism
Describe the most common pharmacologic and mechanical treatments for priapism
Demonstrate appropriate aspiration and injection techniques for managing priapism

## Slide 3
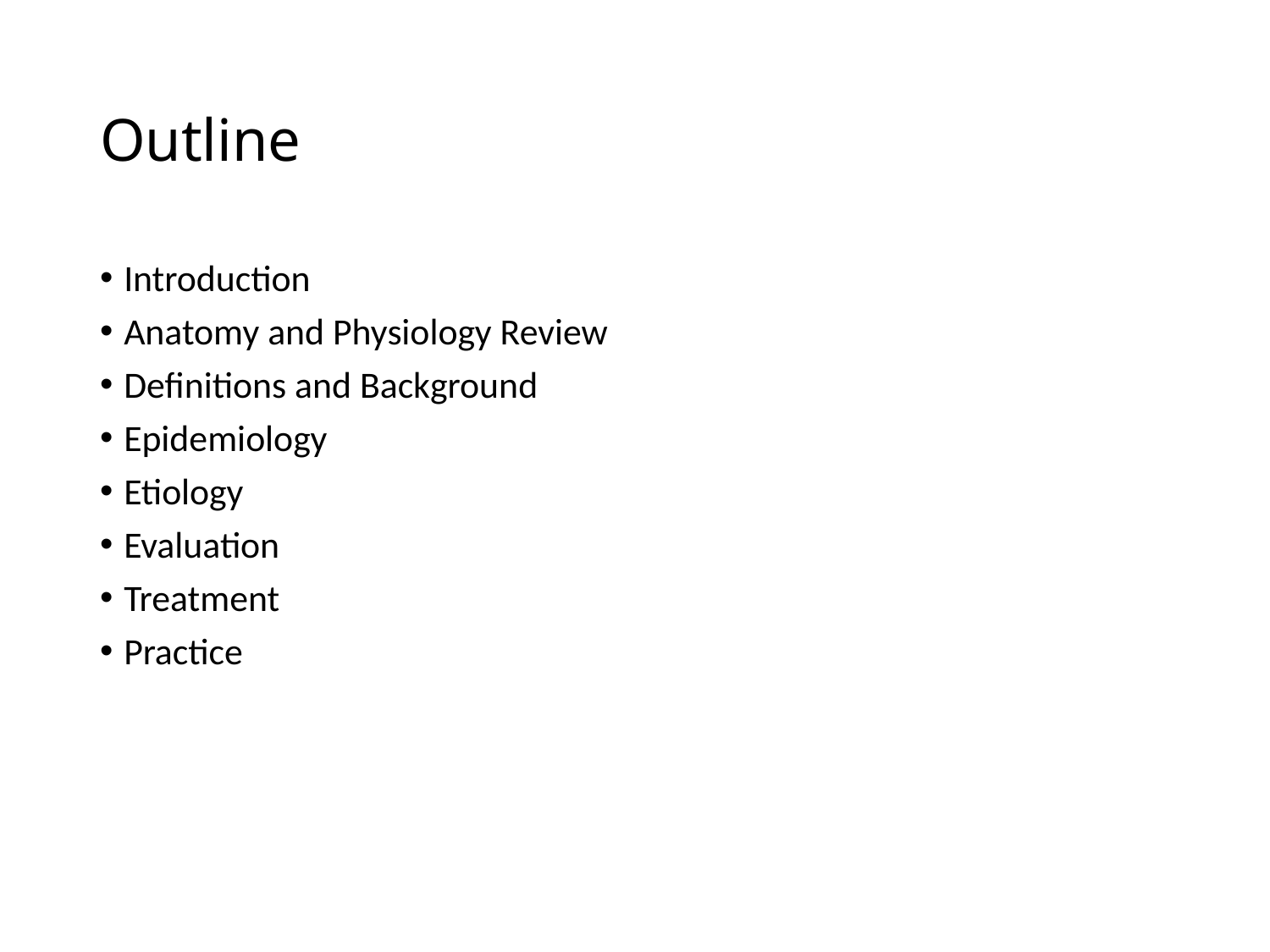

# Outline
Introduction
Anatomy and Physiology Review
Definitions and Background
Epidemiology
Etiology
Evaluation
Treatment
Practice

## Slide 4
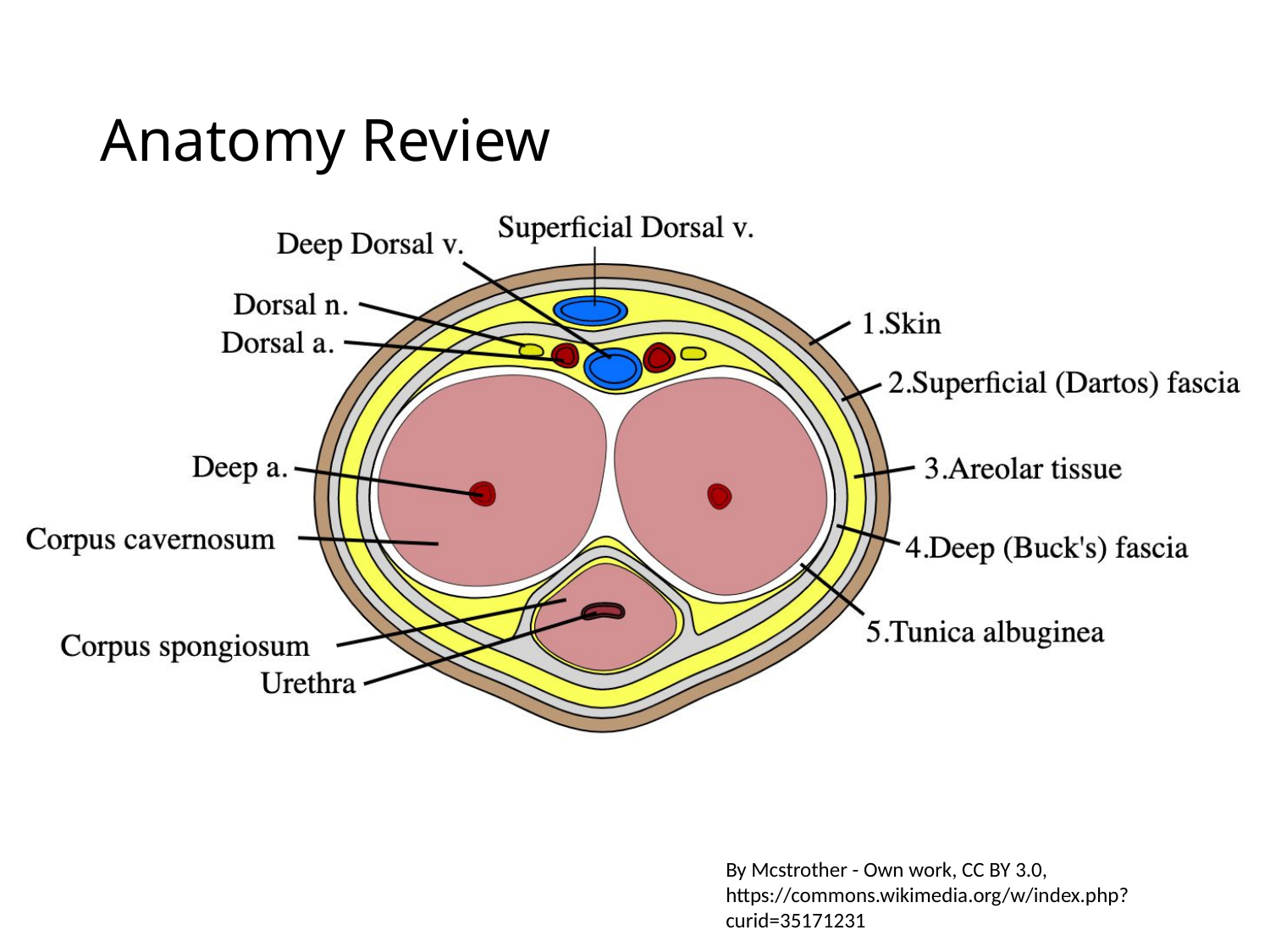

# Anatomy Review
By Mcstrother - Own work, CC BY 3.0, https://commons.wikimedia.org/w/index.php?curid=35171231

## Slide 5
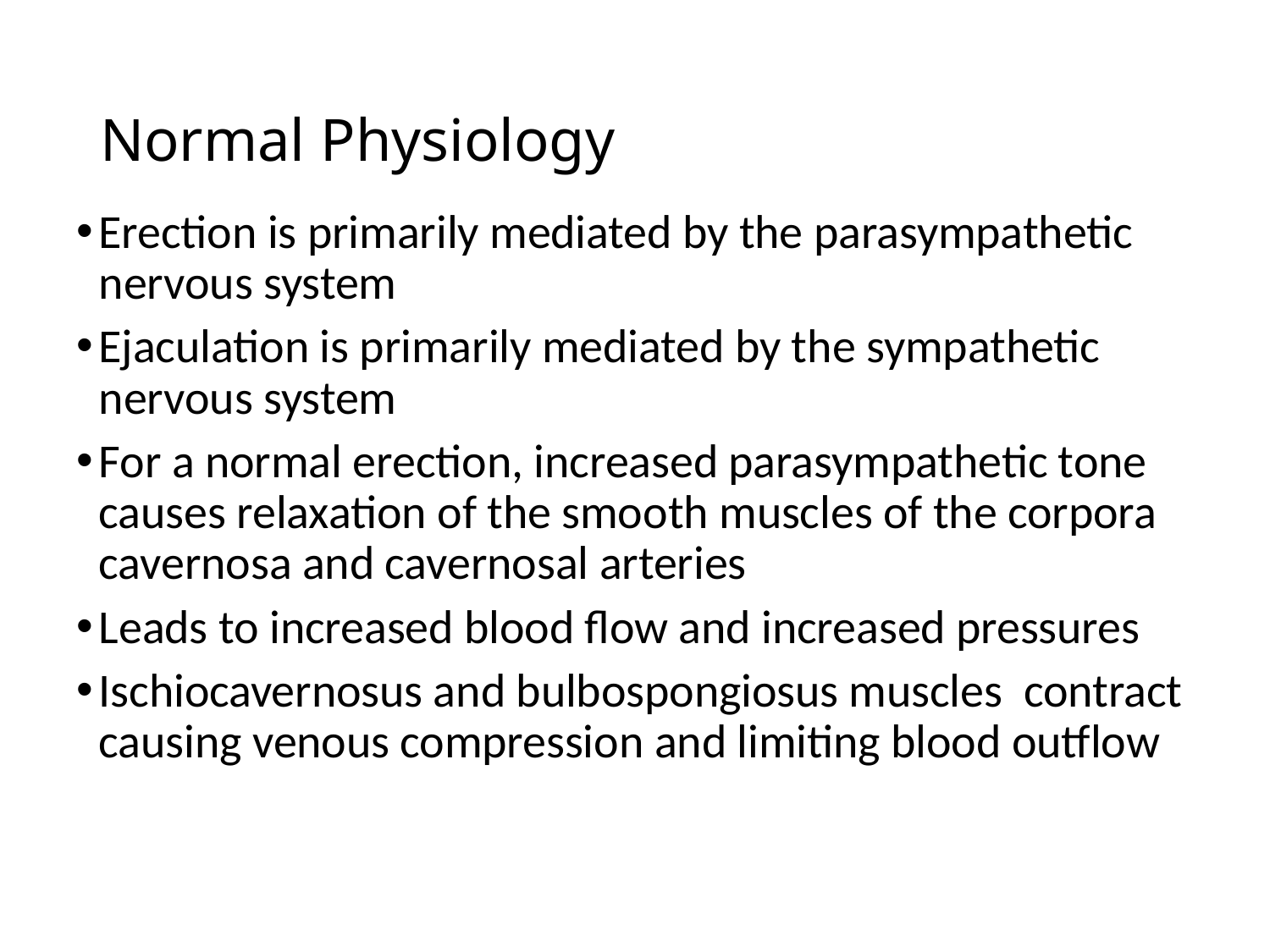

# Normal Physiology
Erection is primarily mediated by the parasympathetic nervous system
Ejaculation is primarily mediated by the sympathetic nervous system
For a normal erection, increased parasympathetic tone causes relaxation of the smooth muscles of the corpora cavernosa and cavernosal arteries
Leads to increased blood flow and increased pressures
Ischiocavernosus and bulbospongiosus muscles contract causing venous compression and limiting blood outflow

## Slide 6
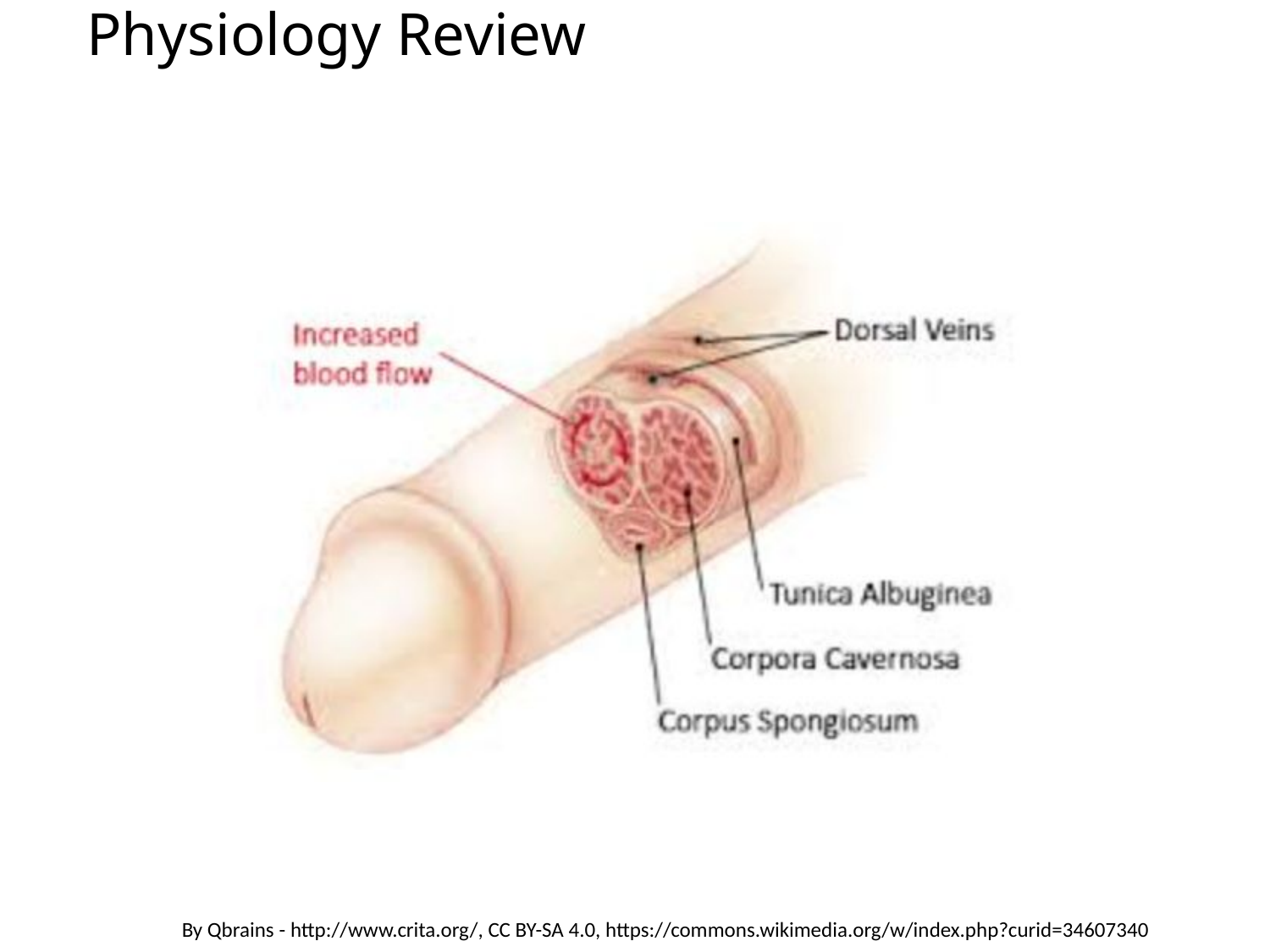

# Physiology Review
By Qbrains - http://www.crita.org/, CC BY-SA 4.0, https://commons.wikimedia.org/w/index.php?curid=34607340

## Slide 7
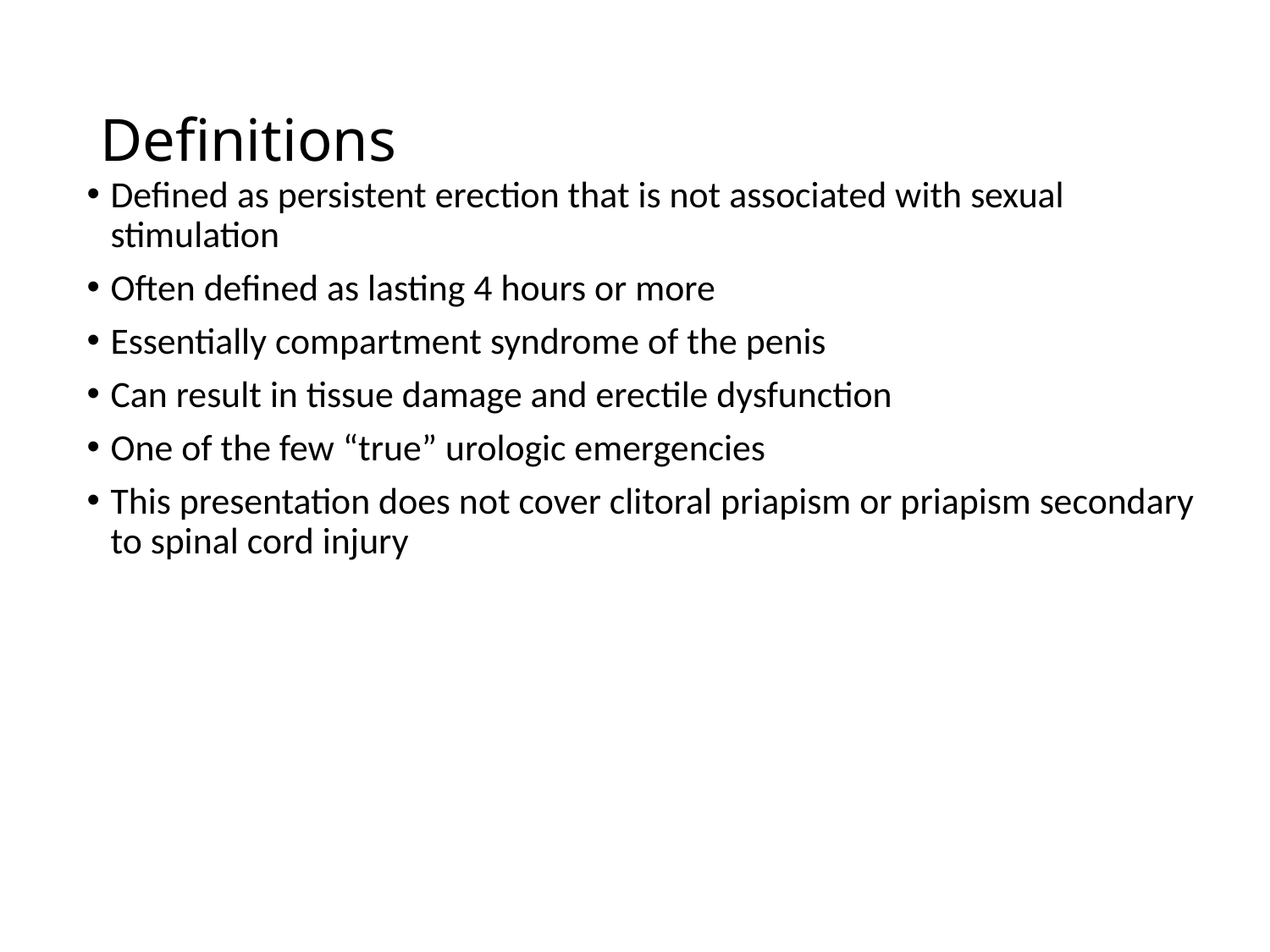

# Definitions
Defined as persistent erection that is not associated with sexual stimulation
Often defined as lasting 4 hours or more
Essentially compartment syndrome of the penis
Can result in tissue damage and erectile dysfunction
One of the few “true” urologic emergencies
This presentation does not cover clitoral priapism or priapism secondary to spinal cord injury

## Slide 8
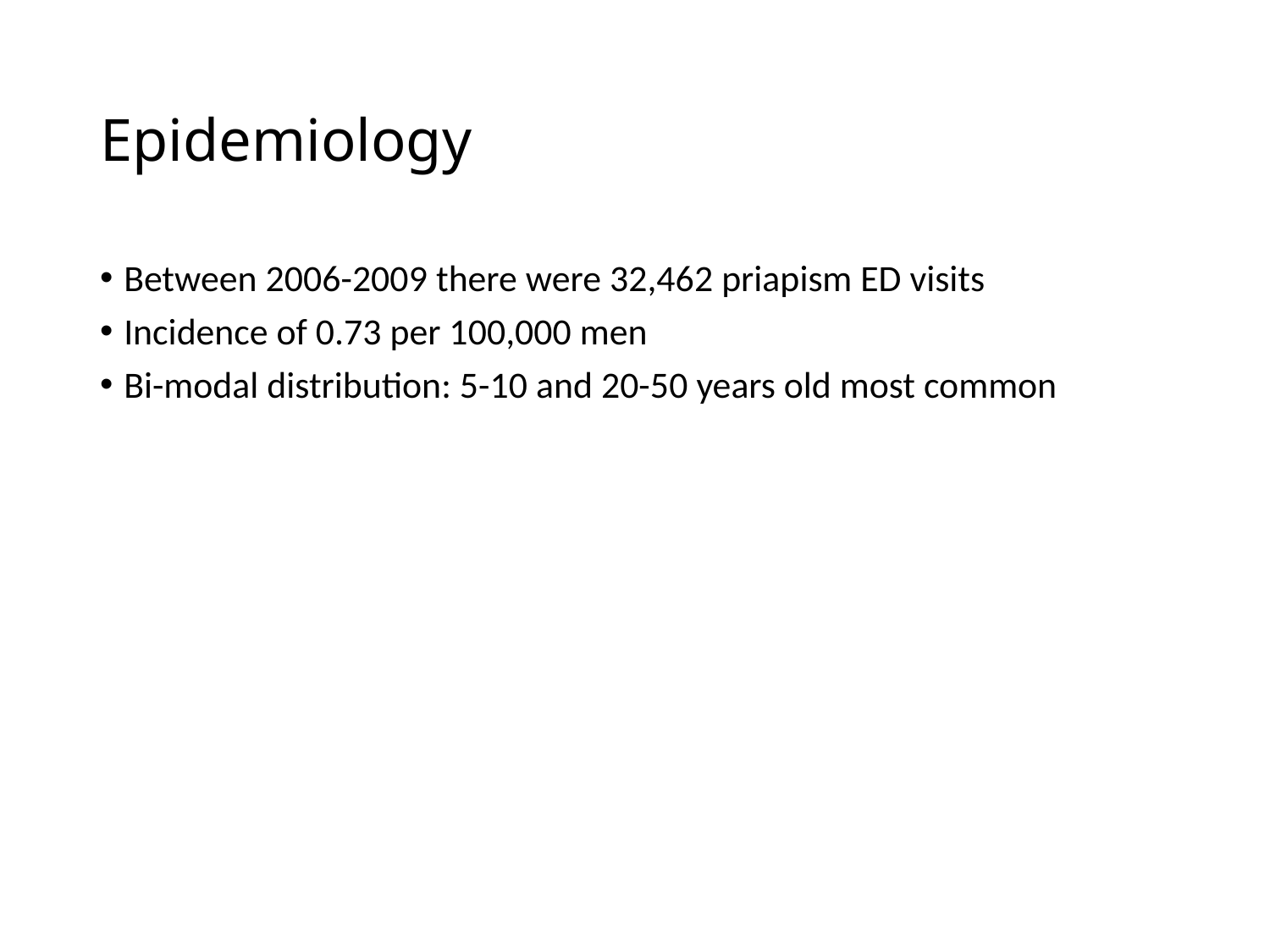

# Epidemiology
Between 2006-2009 there were 32,462 priapism ED visits
Incidence of 0.73 per 100,000 men
Bi-modal distribution: 5-10 and 20-50 years old most common

## Slide 9
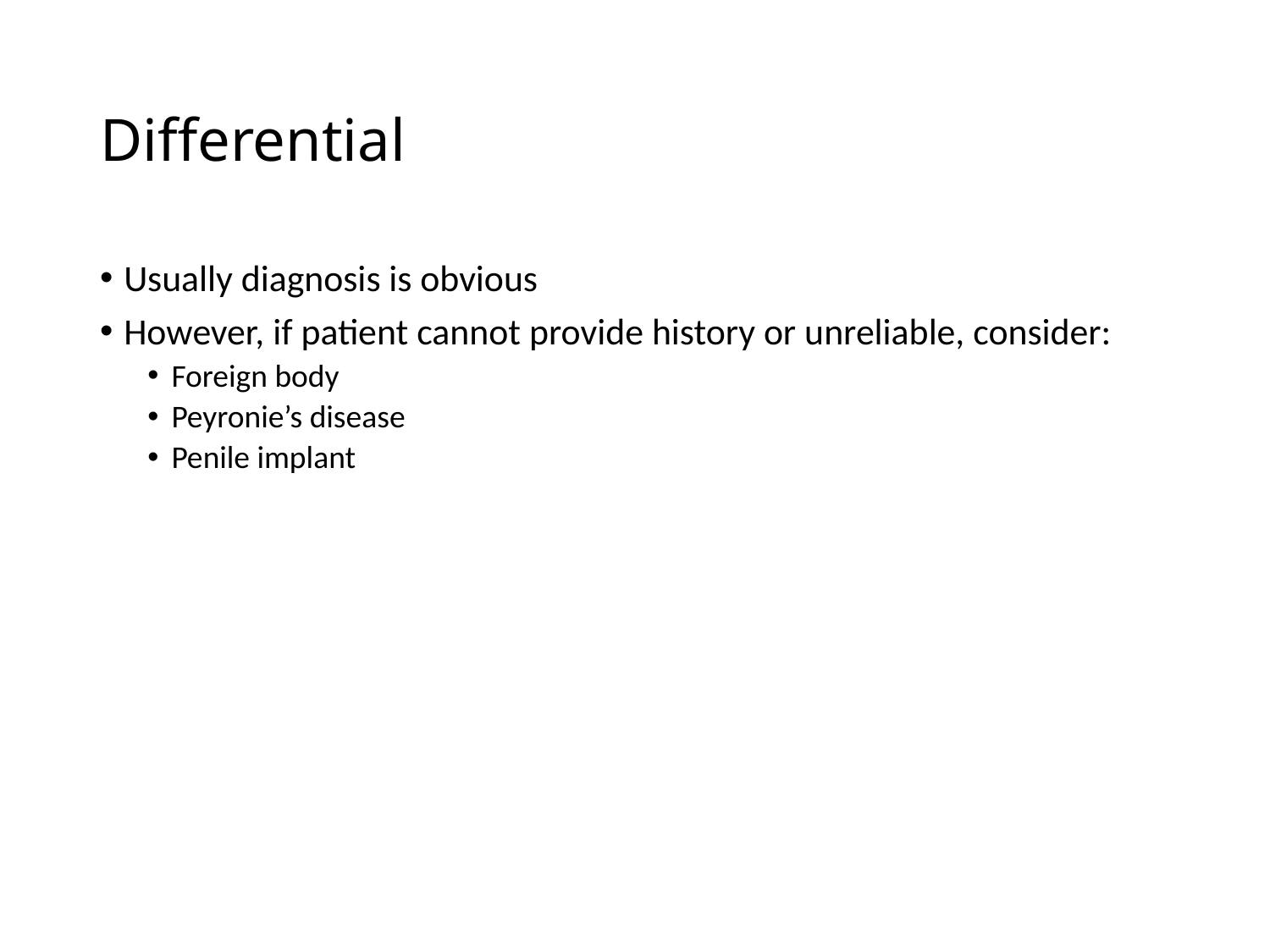

# Differential
Usually diagnosis is obvious
However, if patient cannot provide history or unreliable, consider:
Foreign body
Peyronie’s disease
Penile implant

## Slide 10
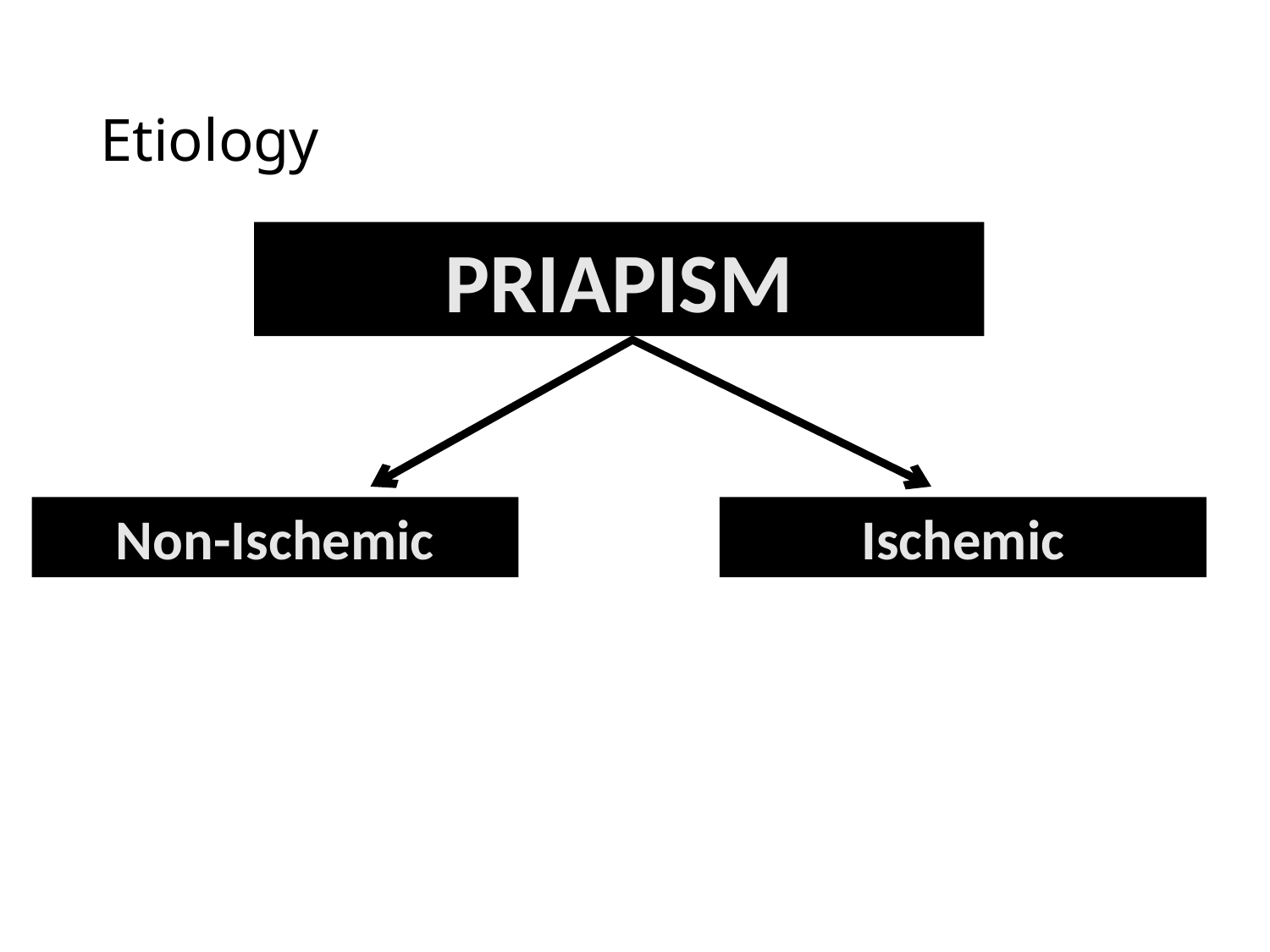

# Etiology
PRIAPISM
Non-Ischemic
Ischemic

## Slide 11
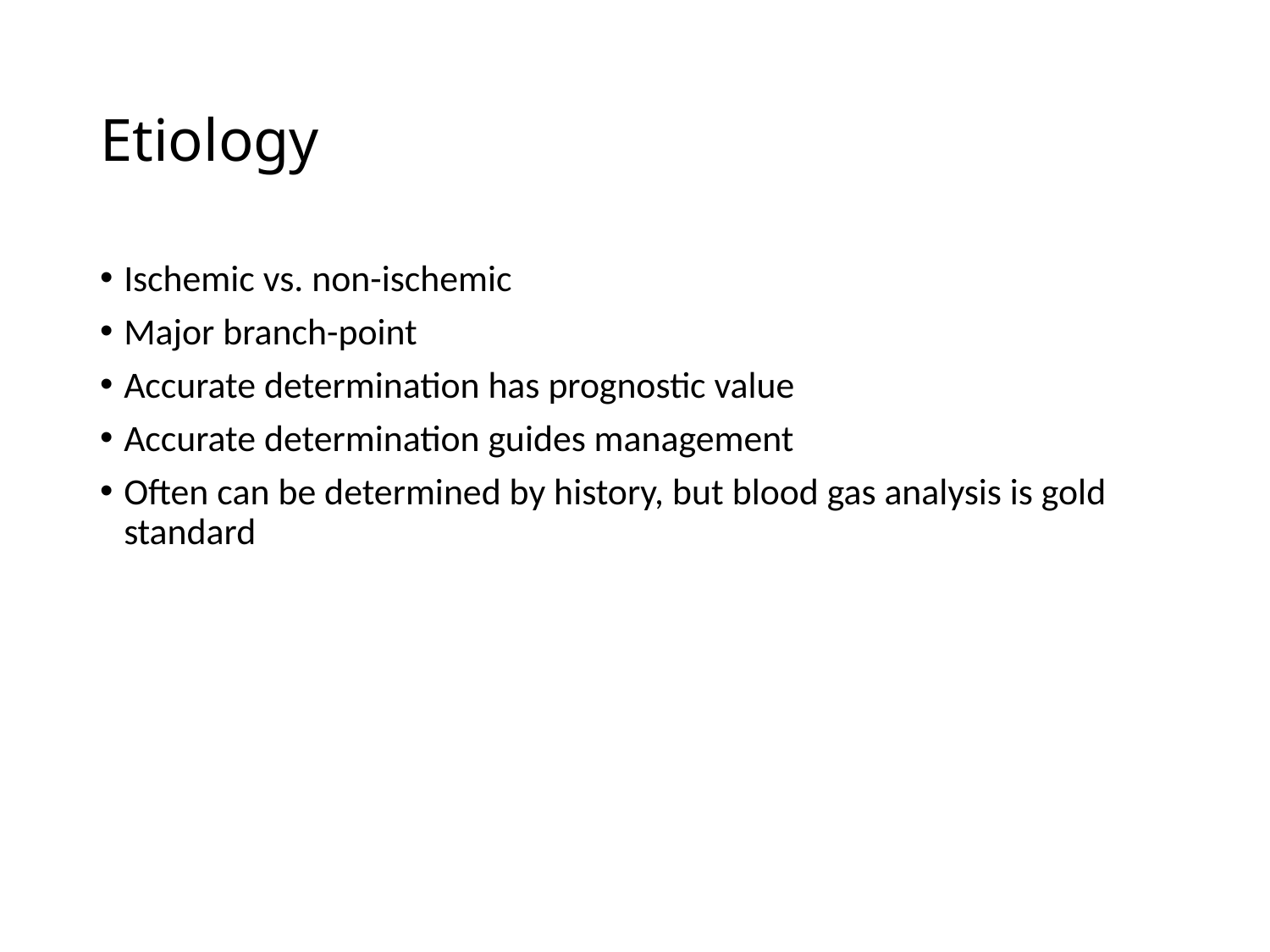

# Etiology
Ischemic vs. non-ischemic
Major branch-point
Accurate determination has prognostic value
Accurate determination guides management
Often can be determined by history, but blood gas analysis is gold standard

## Slide 12
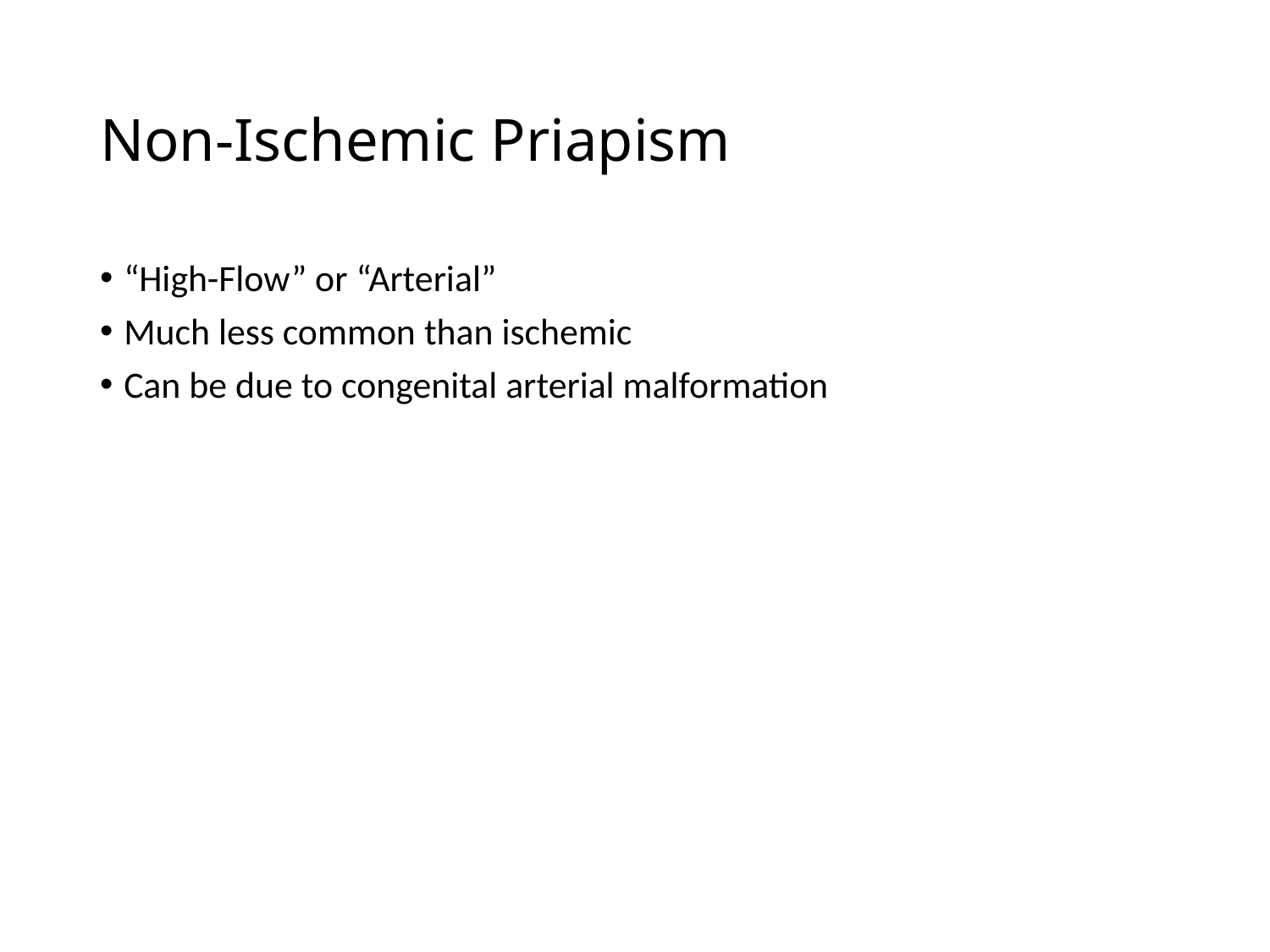

# Non-Ischemic Priapism
“High-Flow” or “Arterial”
Much less common than ischemic
Can be due to congenital arterial malformation

## Slide 13
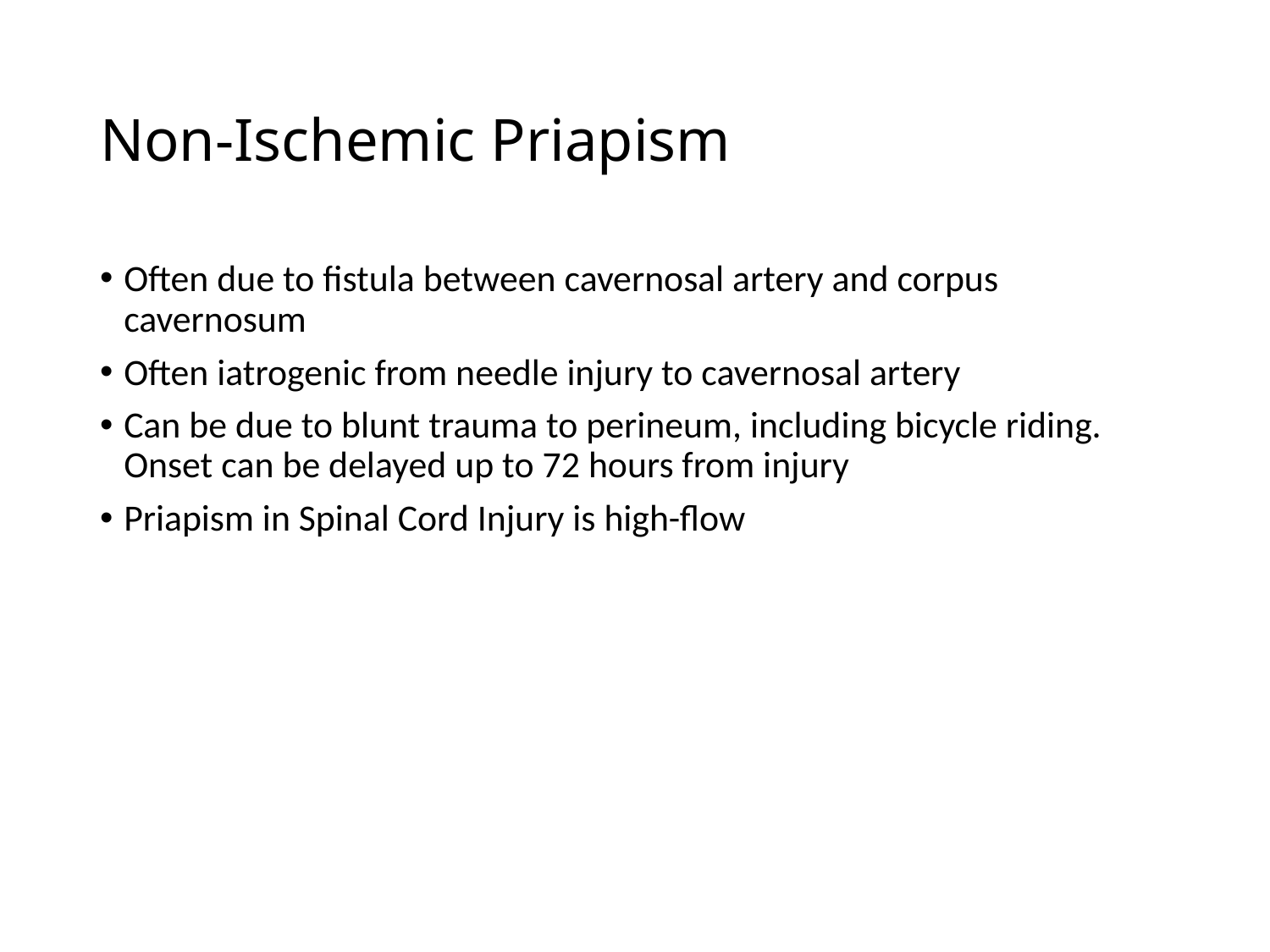

# Non-Ischemic Priapism
Often due to fistula between cavernosal artery and corpus cavernosum
Often iatrogenic from needle injury to cavernosal artery
Can be due to blunt trauma to perineum, including bicycle riding. Onset can be delayed up to 72 hours from injury
Priapism in Spinal Cord Injury is high-flow

## Slide 14
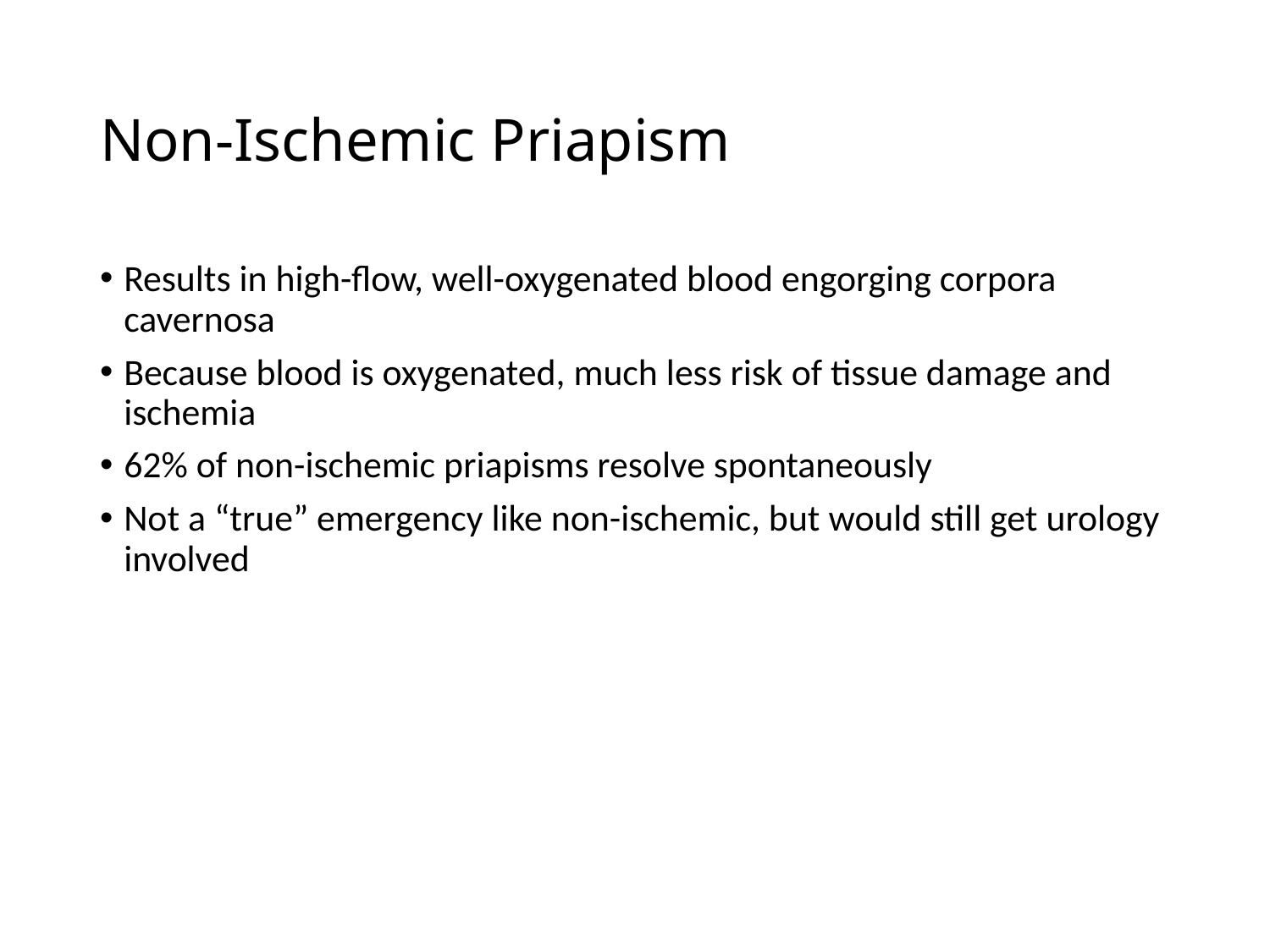

# Non-Ischemic Priapism
Results in high-flow, well-oxygenated blood engorging corpora cavernosa
Because blood is oxygenated, much less risk of tissue damage and ischemia
62% of non-ischemic priapisms resolve spontaneously
Not a “true” emergency like non-ischemic, but would still get urology involved

## Slide 15
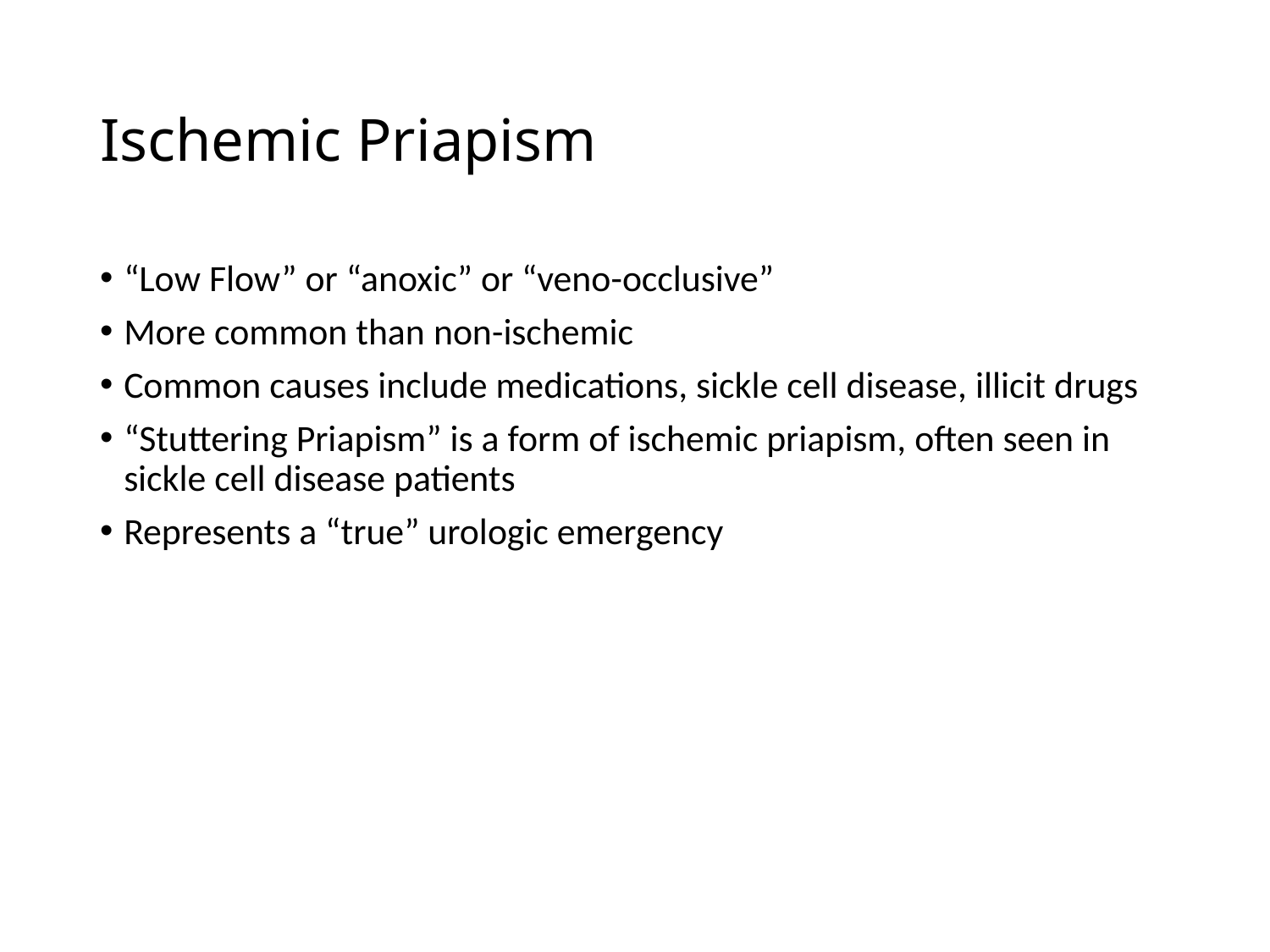

# Ischemic Priapism
“Low Flow” or “anoxic” or “veno-occlusive”
More common than non-ischemic
Common causes include medications, sickle cell disease, illicit drugs
“Stuttering Priapism” is a form of ischemic priapism, often seen in sickle cell disease patients
Represents a “true” urologic emergency

## Slide 16
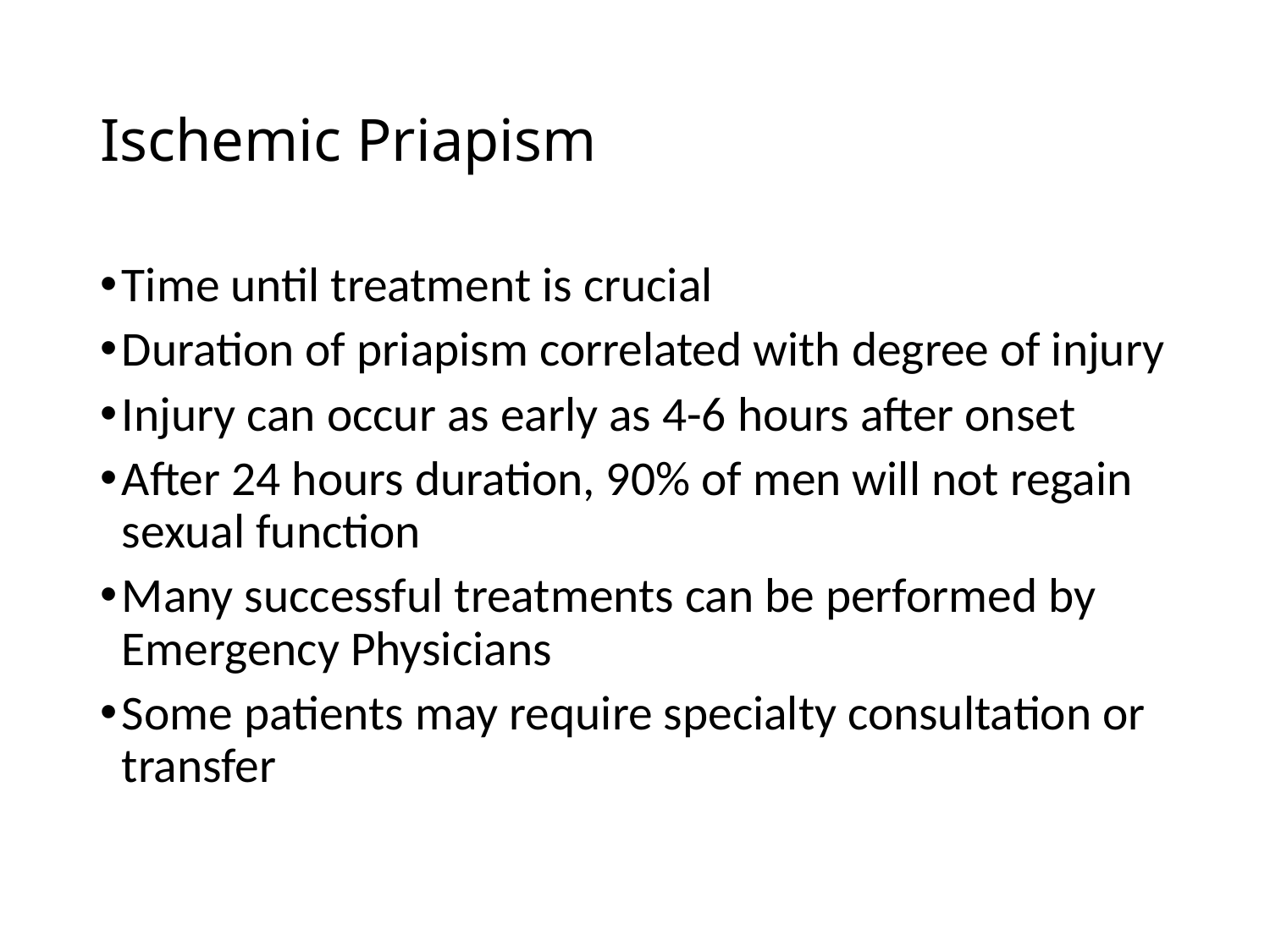

# Ischemic Priapism
Time until treatment is crucial
Duration of priapism correlated with degree of injury
Injury can occur as early as 4-6 hours after onset
After 24 hours duration, 90% of men will not regain sexual function
Many successful treatments can be performed by Emergency Physicians
Some patients may require specialty consultation or transfer

## Slide 17
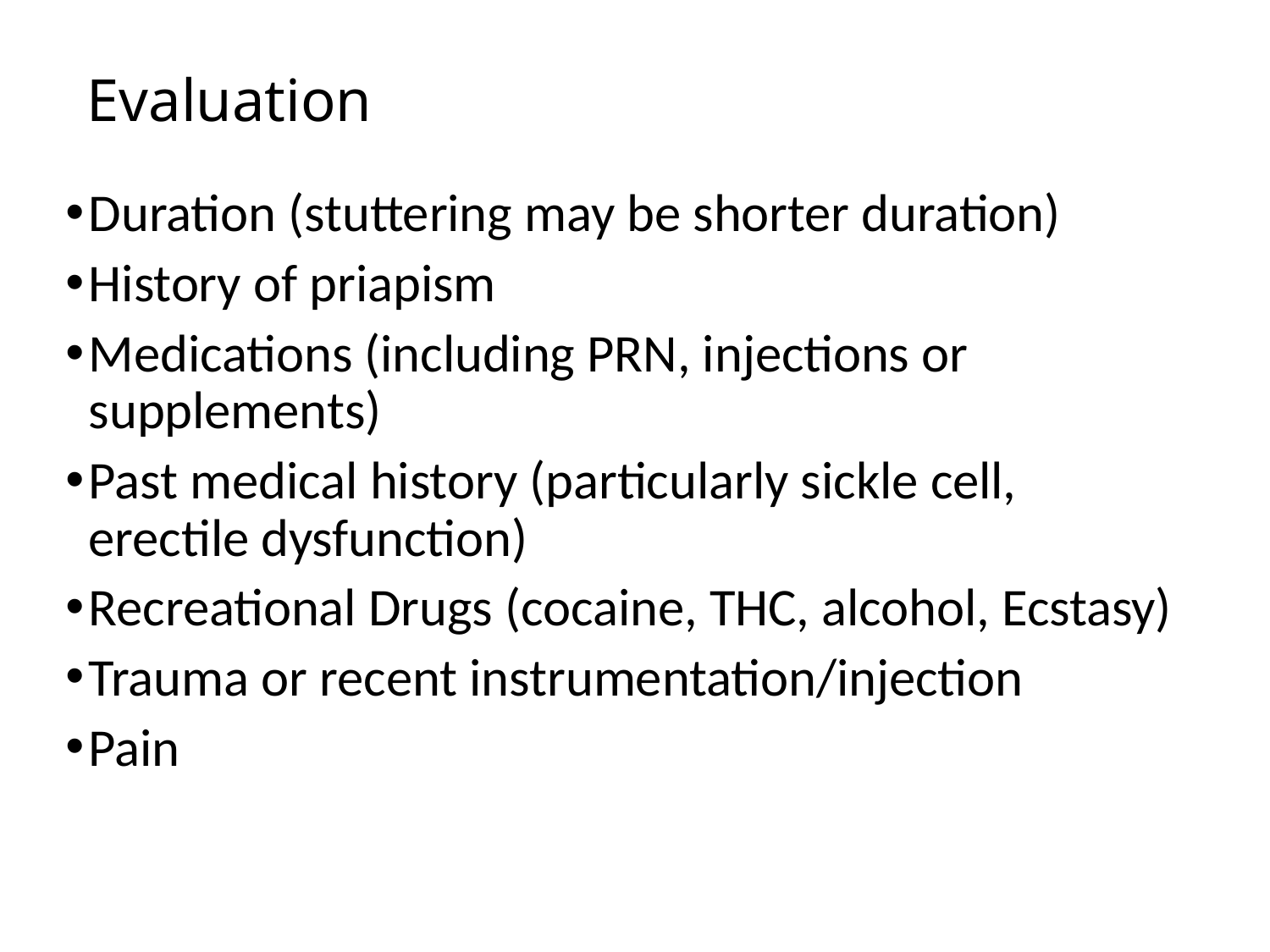

# Evaluation
Duration (stuttering may be shorter duration)
History of priapism
Medications (including PRN, injections or supplements)
Past medical history (particularly sickle cell, erectile dysfunction)
Recreational Drugs (cocaine, THC, alcohol, Ecstasy)
Trauma or recent instrumentation/injection
Pain

## Slide 18
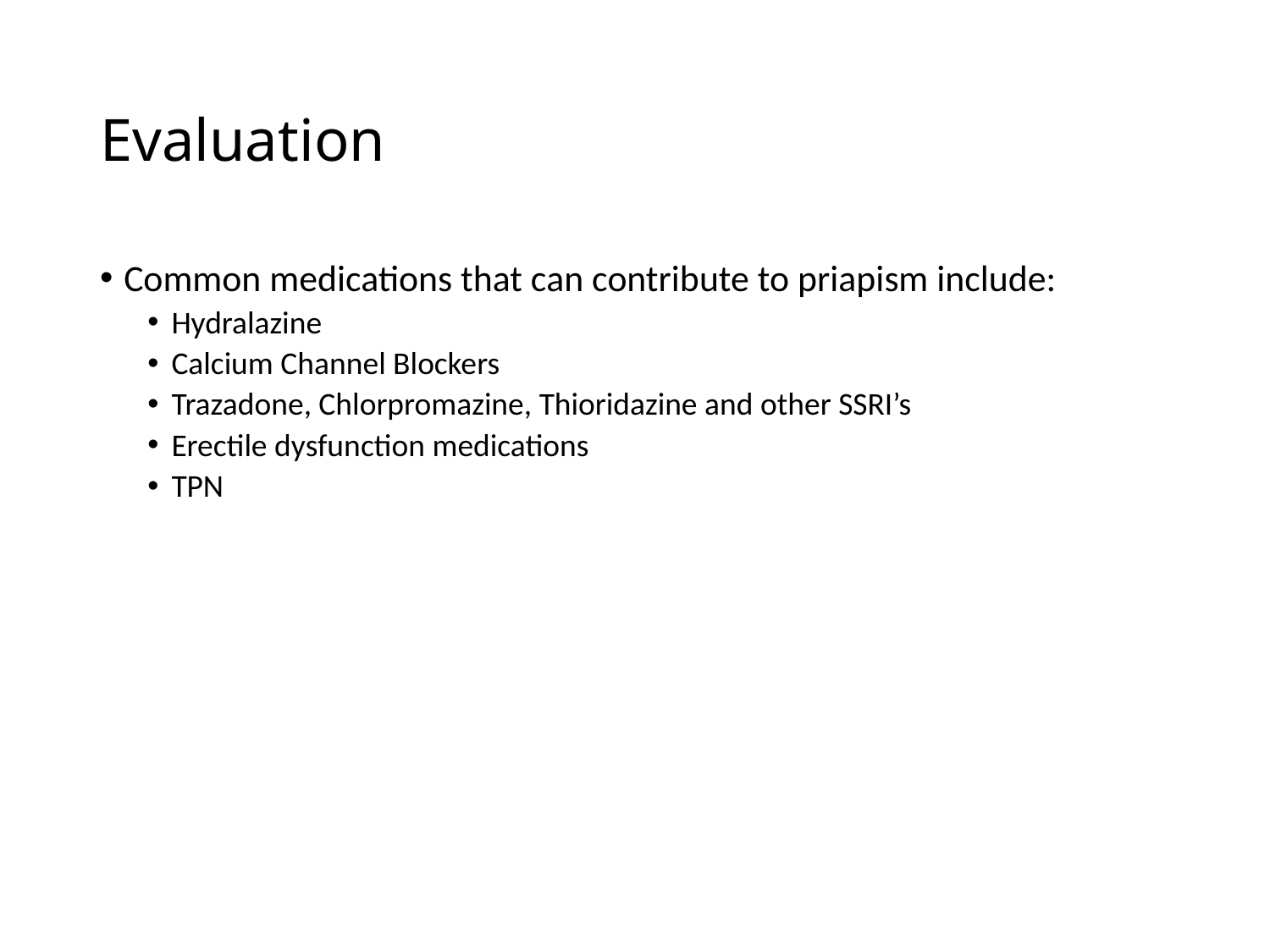

# Evaluation
Common medications that can contribute to priapism include:
Hydralazine
Calcium Channel Blockers
Trazadone, Chlorpromazine, Thioridazine and other SSRI’s
Erectile dysfunction medications
TPN

## Slide 19
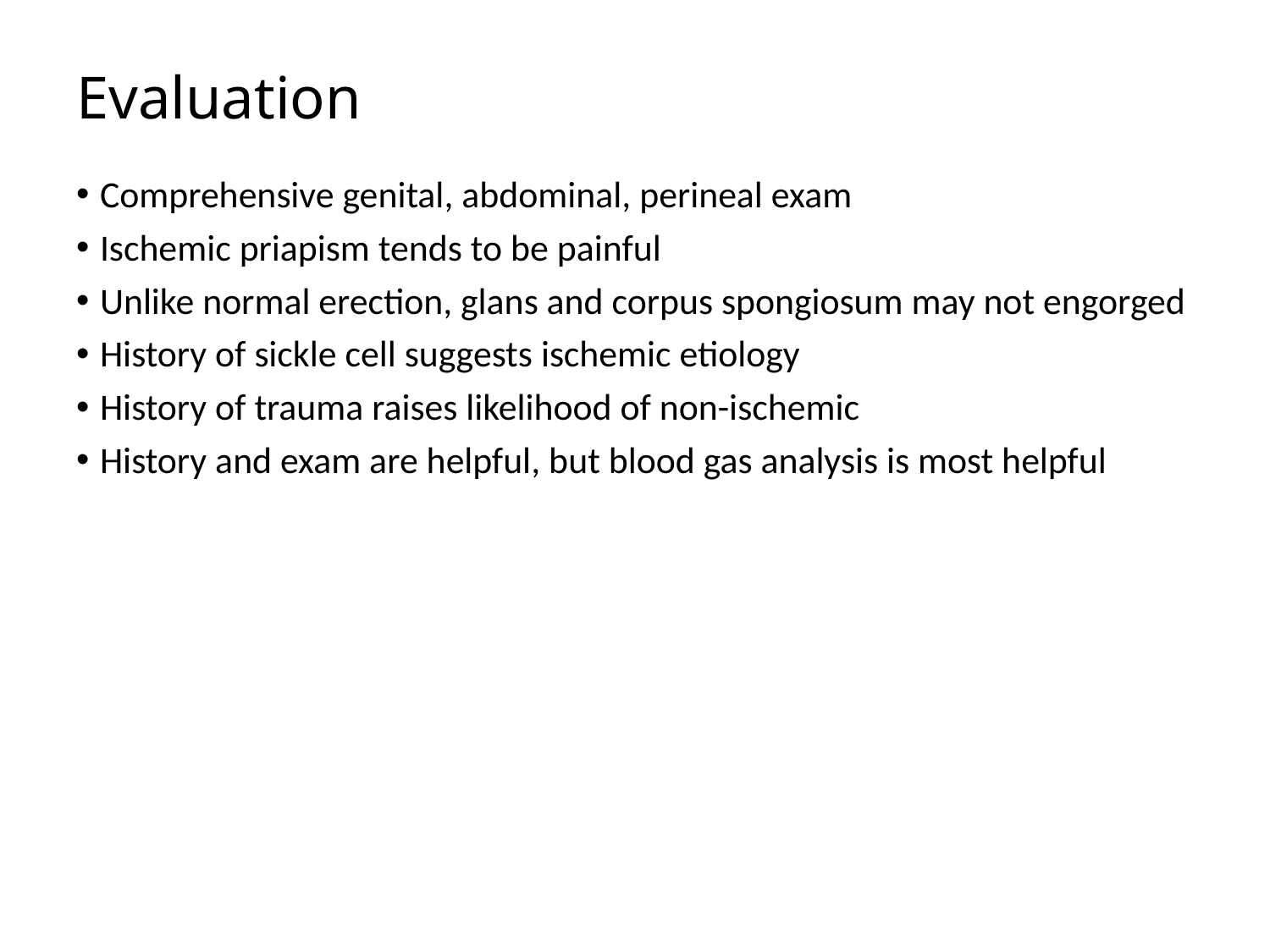

# Evaluation
Comprehensive genital, abdominal, perineal exam
Ischemic priapism tends to be painful
Unlike normal erection, glans and corpus spongiosum may not engorged
History of sickle cell suggests ischemic etiology
History of trauma raises likelihood of non-ischemic
History and exam are helpful, but blood gas analysis is most helpful

## Slide 20
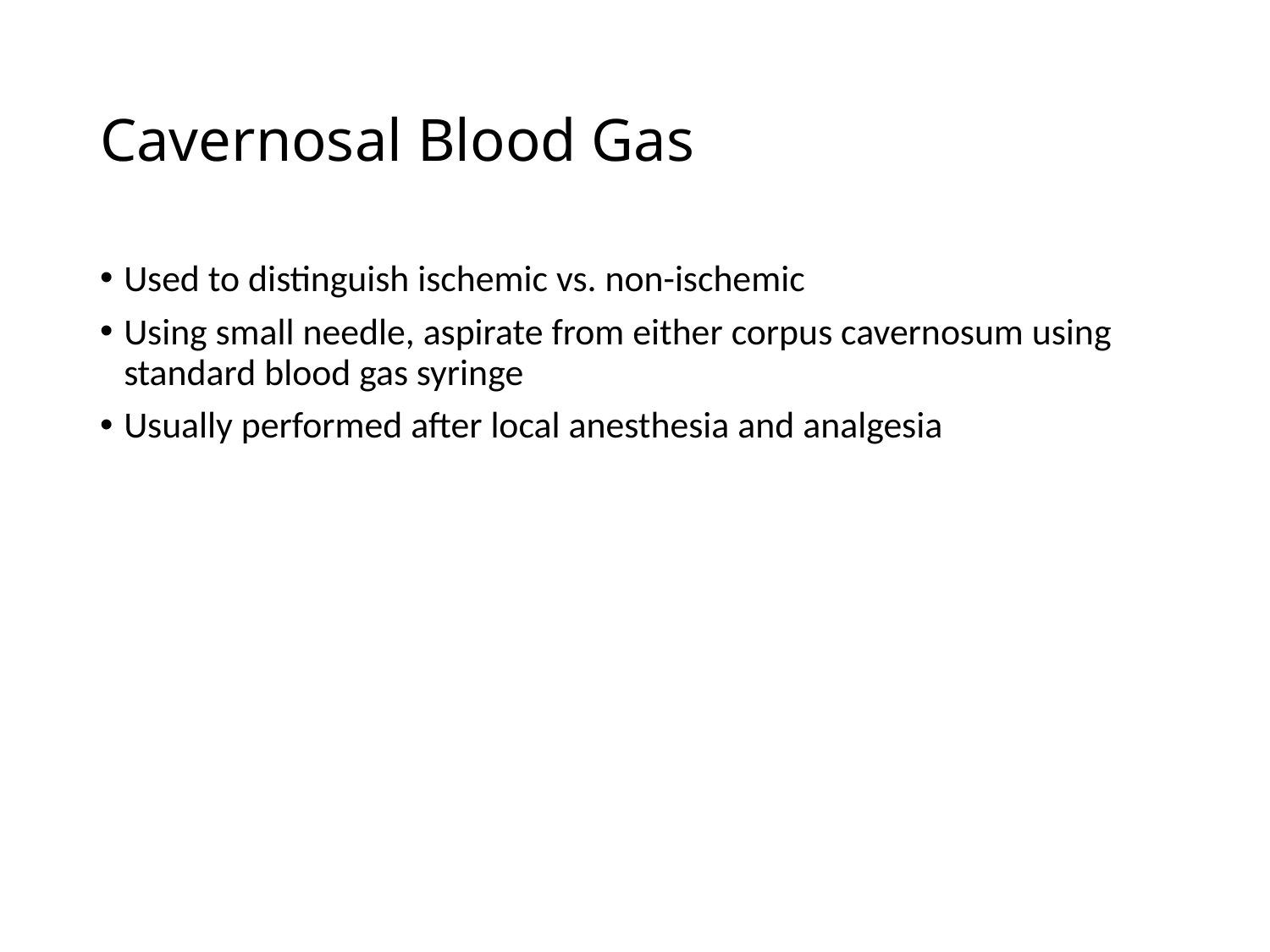

# Cavernosal Blood Gas
Used to distinguish ischemic vs. non-ischemic
Using small needle, aspirate from either corpus cavernosum using standard blood gas syringe
Usually performed after local anesthesia and analgesia

## Slide 21
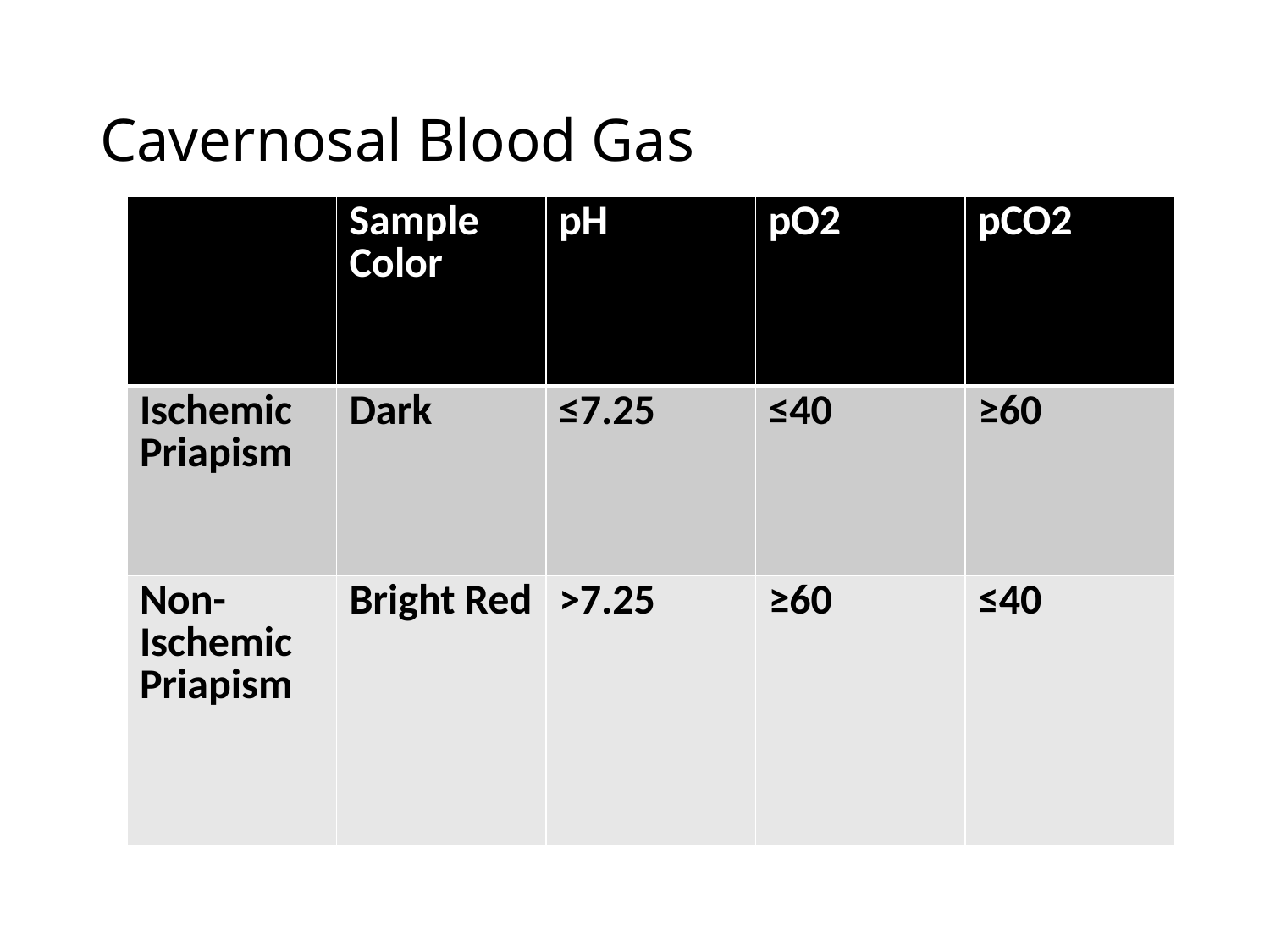

# Cavernosal Blood Gas
| | Sample Color | pH | pO2 | pCO2 |
| --- | --- | --- | --- | --- |
| Ischemic Priapism | Dark | ≤7.25 | ≤40 | ≥60 |
| Non-Ischemic Priapism | Bright Red | >7.25 | ≥60 | ≤40 |

## Slide 22
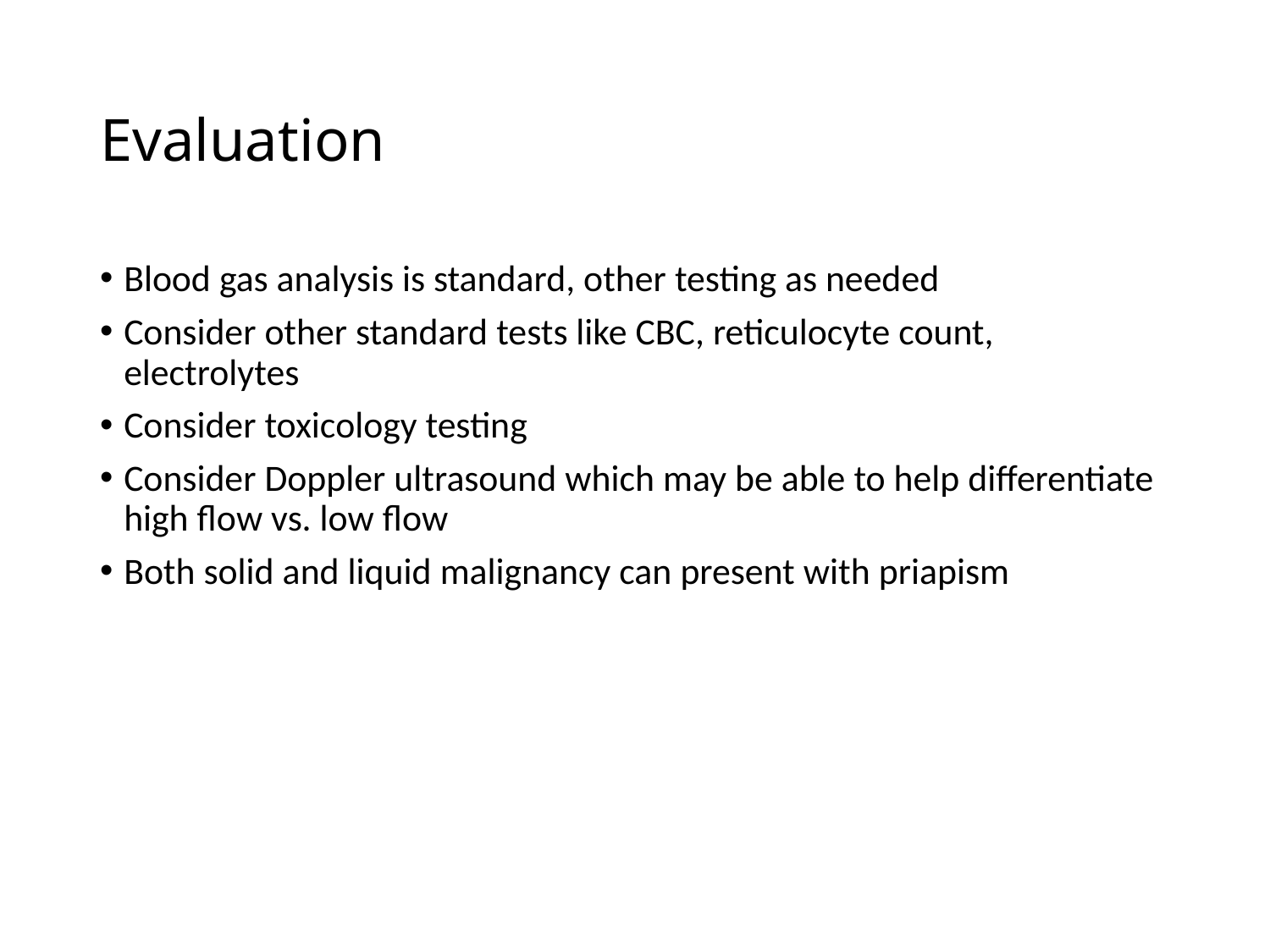

# Evaluation
Blood gas analysis is standard, other testing as needed
Consider other standard tests like CBC, reticulocyte count, electrolytes
Consider toxicology testing
Consider Doppler ultrasound which may be able to help differentiate high flow vs. low flow
Both solid and liquid malignancy can present with priapism

## Slide 23
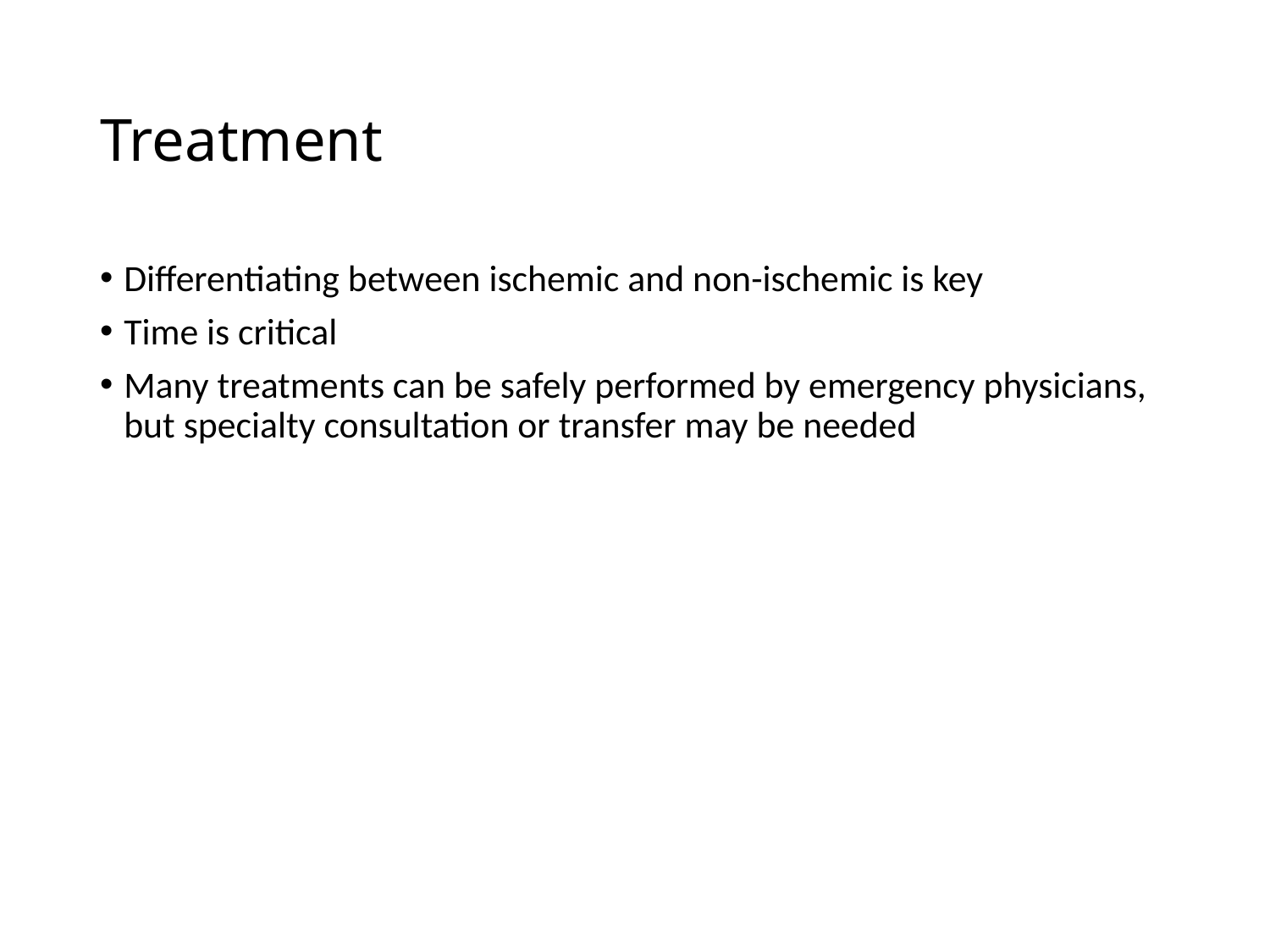

# Treatment
Differentiating between ischemic and non-ischemic is key
Time is critical
Many treatments can be safely performed by emergency physicians, but specialty consultation or transfer may be needed

## Slide 24
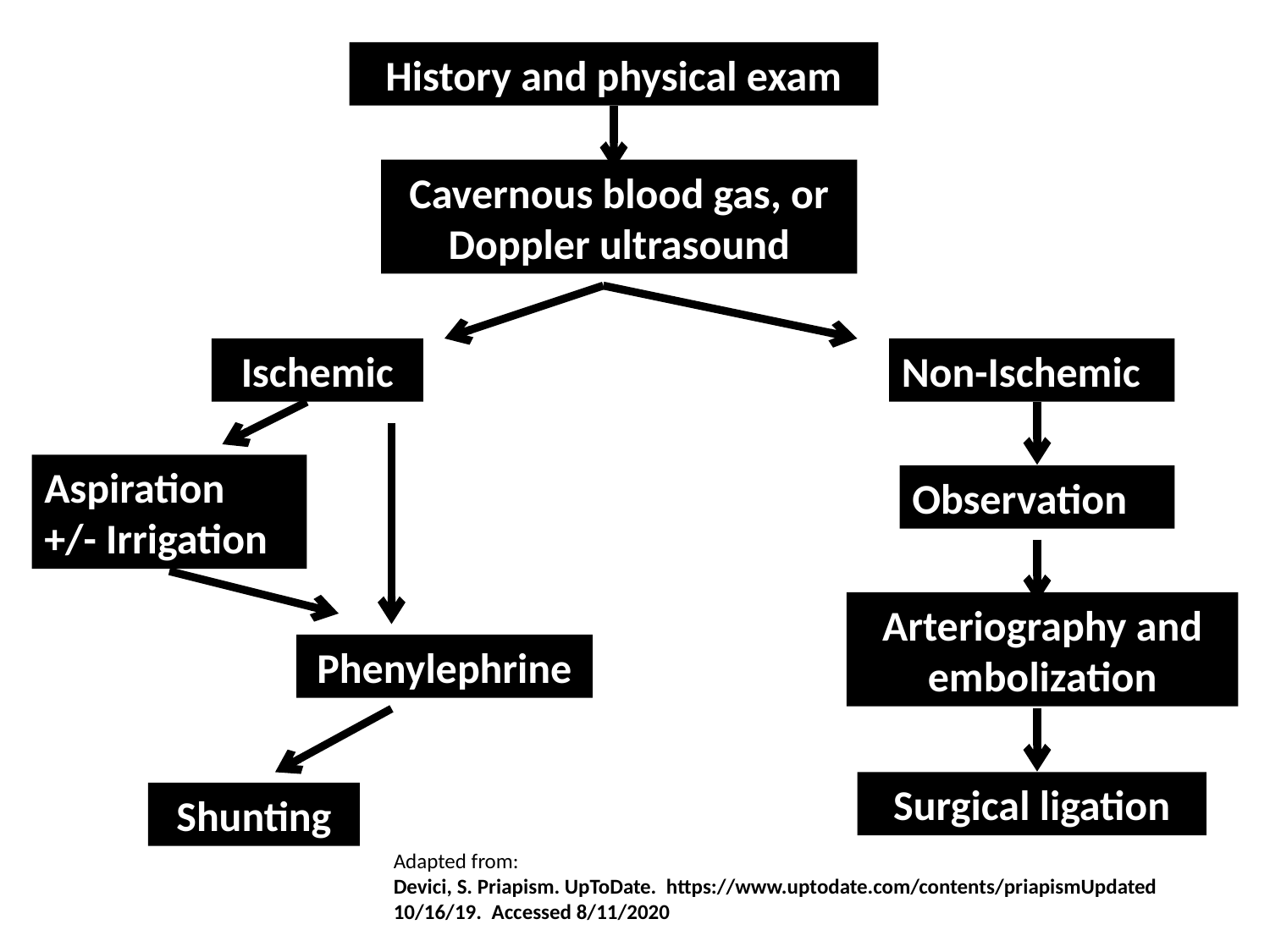

History and physical exam
Cavernous blood gas, or Doppler ultrasound
Ischemic
Non-Ischemic
Aspiration
+/- Irrigation
Observation
Arteriography and embolization
Phenylephrine
Surgical ligation
Shunting
Adapted from:
Devici, S. Priapism. UpToDate. https://www.uptodate.com/contents/priapismUpdated 10/16/19. Accessed 8/11/2020

## Slide 25
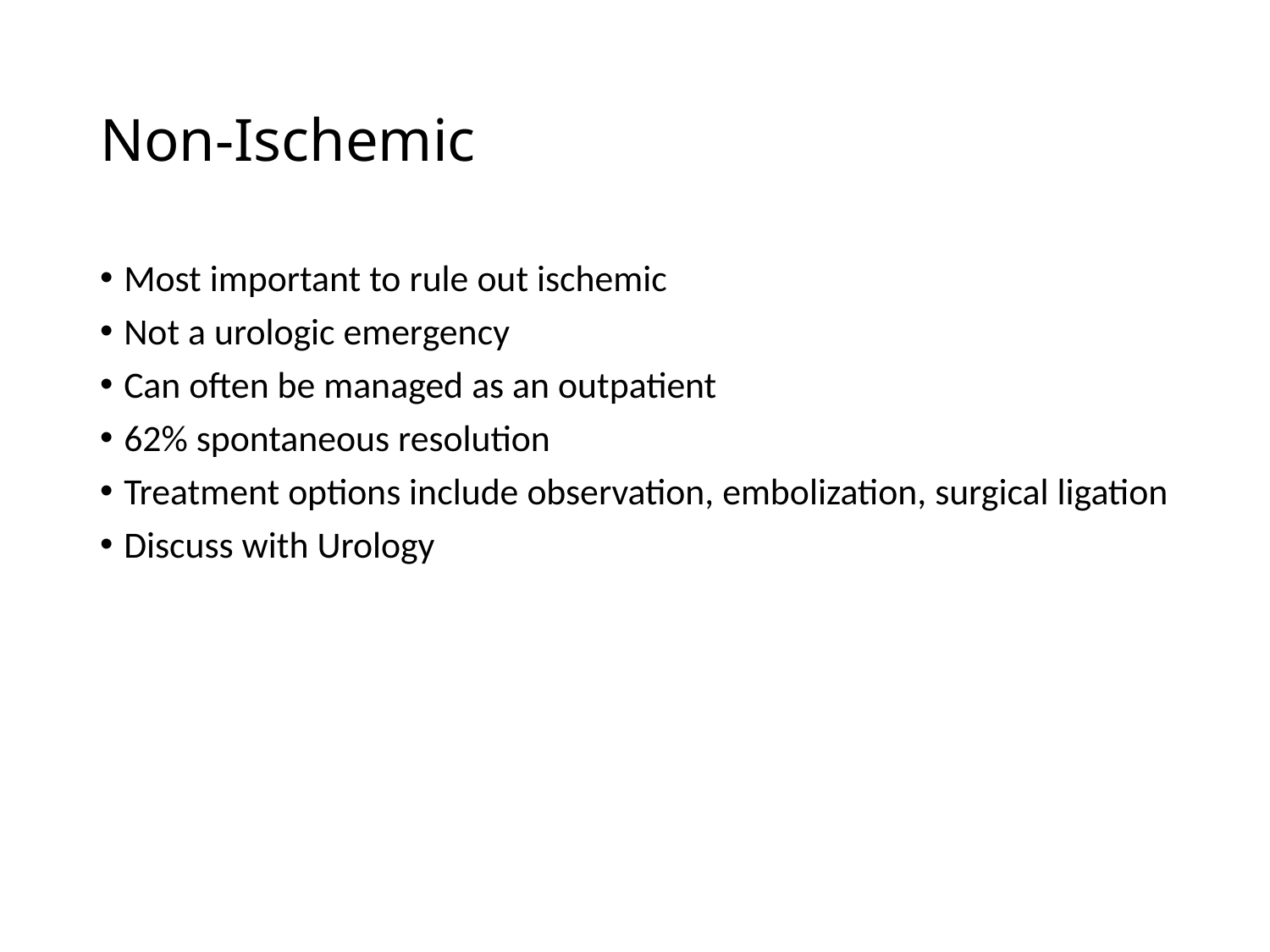

# Non-Ischemic
Most important to rule out ischemic
Not a urologic emergency
Can often be managed as an outpatient
62% spontaneous resolution
Treatment options include observation, embolization, surgical ligation
Discuss with Urology

## Slide 26
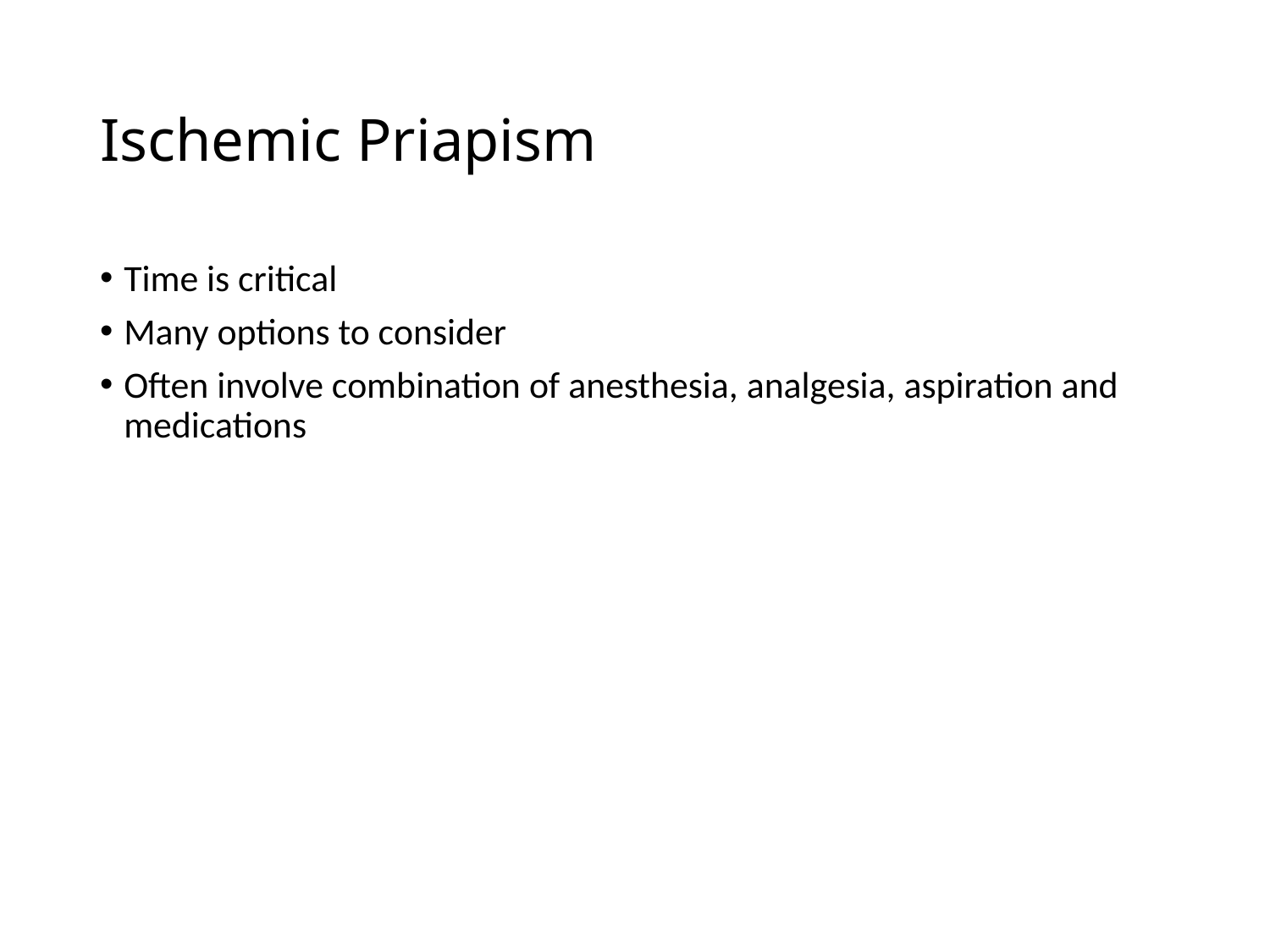

# Ischemic Priapism
Time is critical
Many options to consider
Often involve combination of anesthesia, analgesia, aspiration and medications

## Slide 27
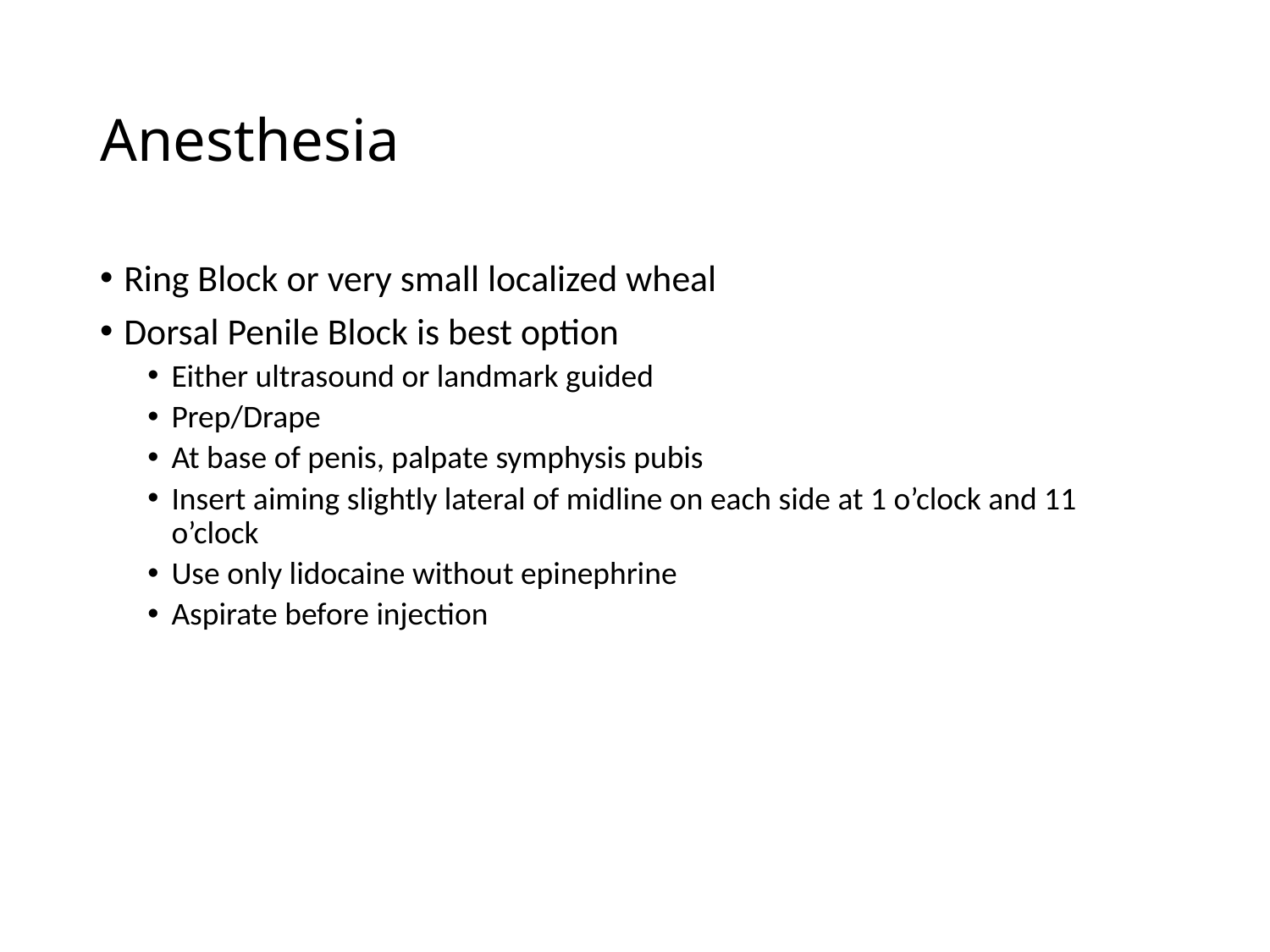

# Anesthesia
Ring Block or very small localized wheal
Dorsal Penile Block is best option
Either ultrasound or landmark guided
Prep/Drape
At base of penis, palpate symphysis pubis
Insert aiming slightly lateral of midline on each side at 1 o’clock and 11 o’clock
Use only lidocaine without epinephrine
Aspirate before injection

## Slide 28
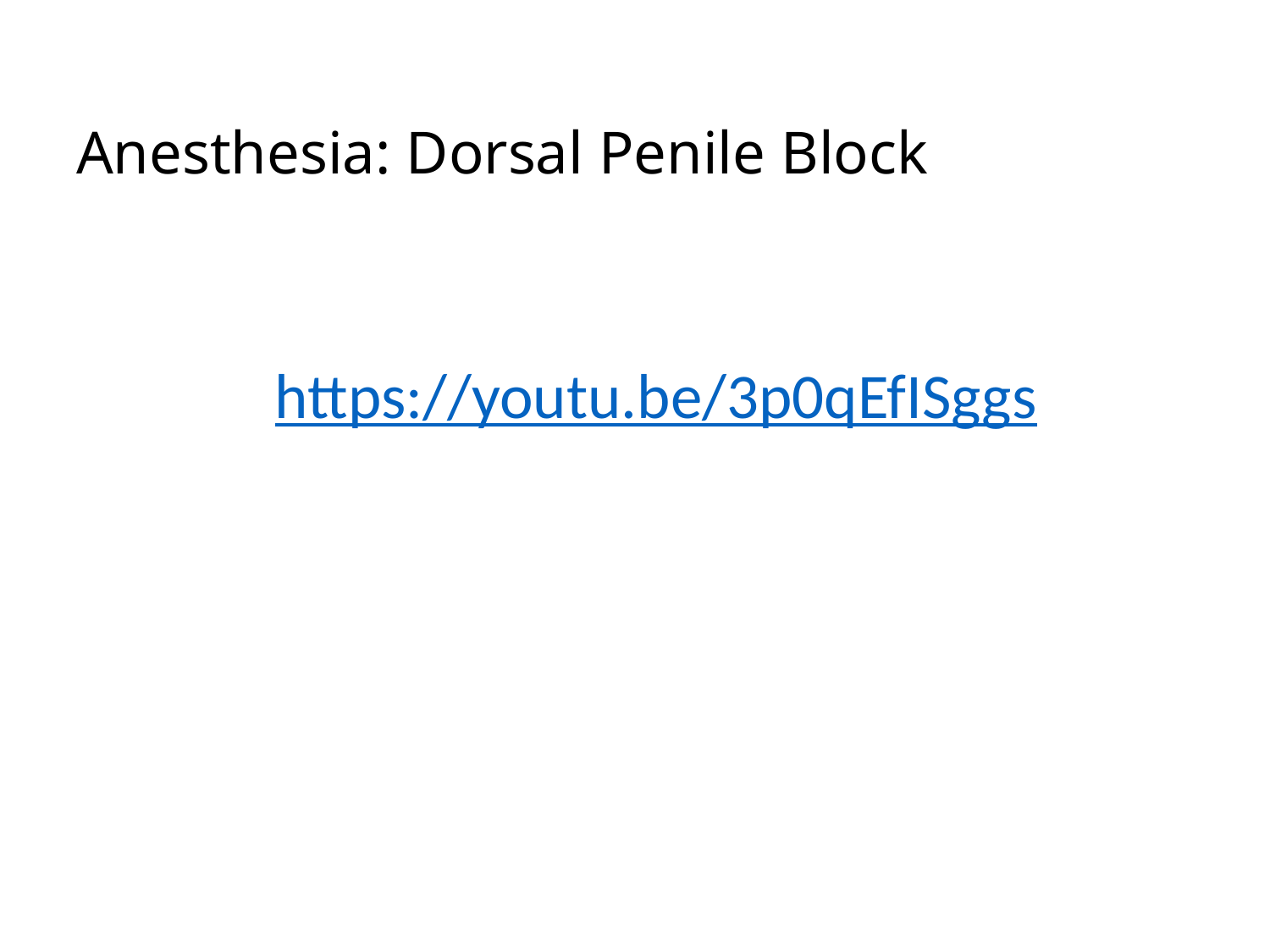

# Anesthesia: Dorsal Penile Block
https://youtu.be/3p0qEfISggs

## Slide 29
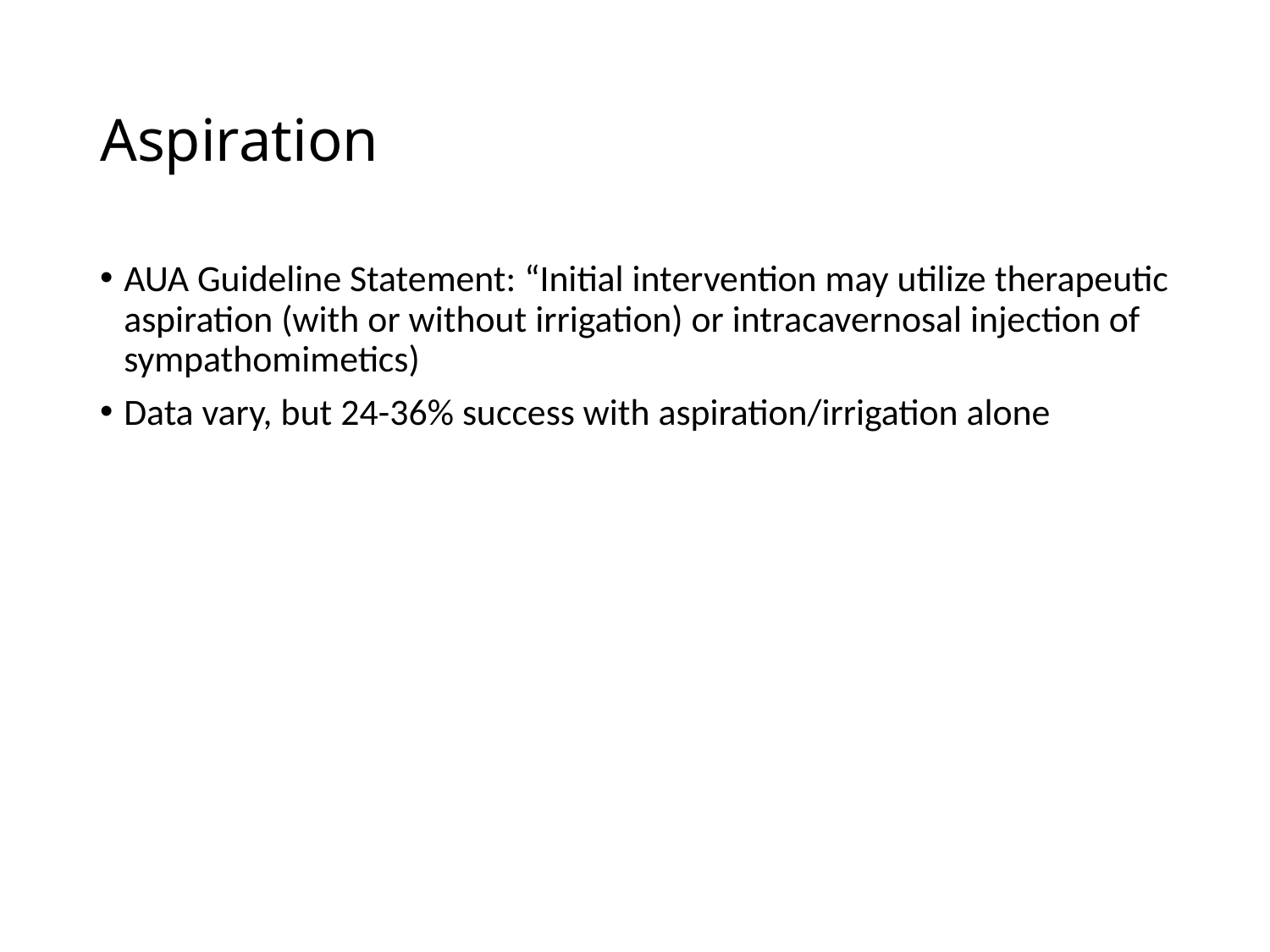

# Aspiration
AUA Guideline Statement: “Initial intervention may utilize therapeutic aspiration (with or without irrigation) or intracavernosal injection of sympathomimetics)
Data vary, but 24-36% success with aspiration/irrigation alone

## Slide 30
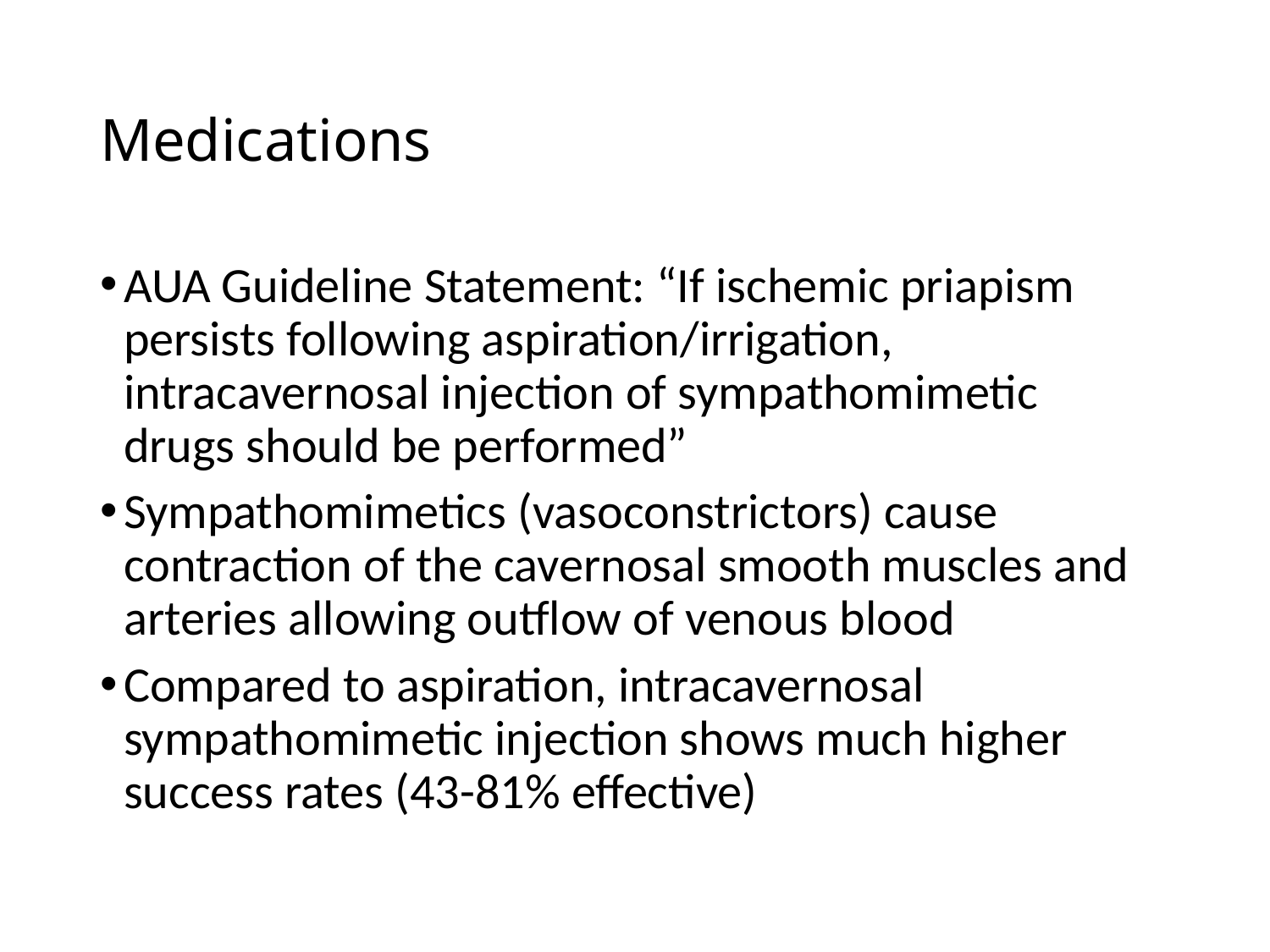

# Medications
AUA Guideline Statement: “If ischemic priapism persists following aspiration/irrigation, intracavernosal injection of sympathomimetic drugs should be performed”
Sympathomimetics (vasoconstrictors) cause contraction of the cavernosal smooth muscles and arteries allowing outflow of venous blood
Compared to aspiration, intracavernosal sympathomimetic injection shows much higher success rates (43-81% effective)

## Slide 31
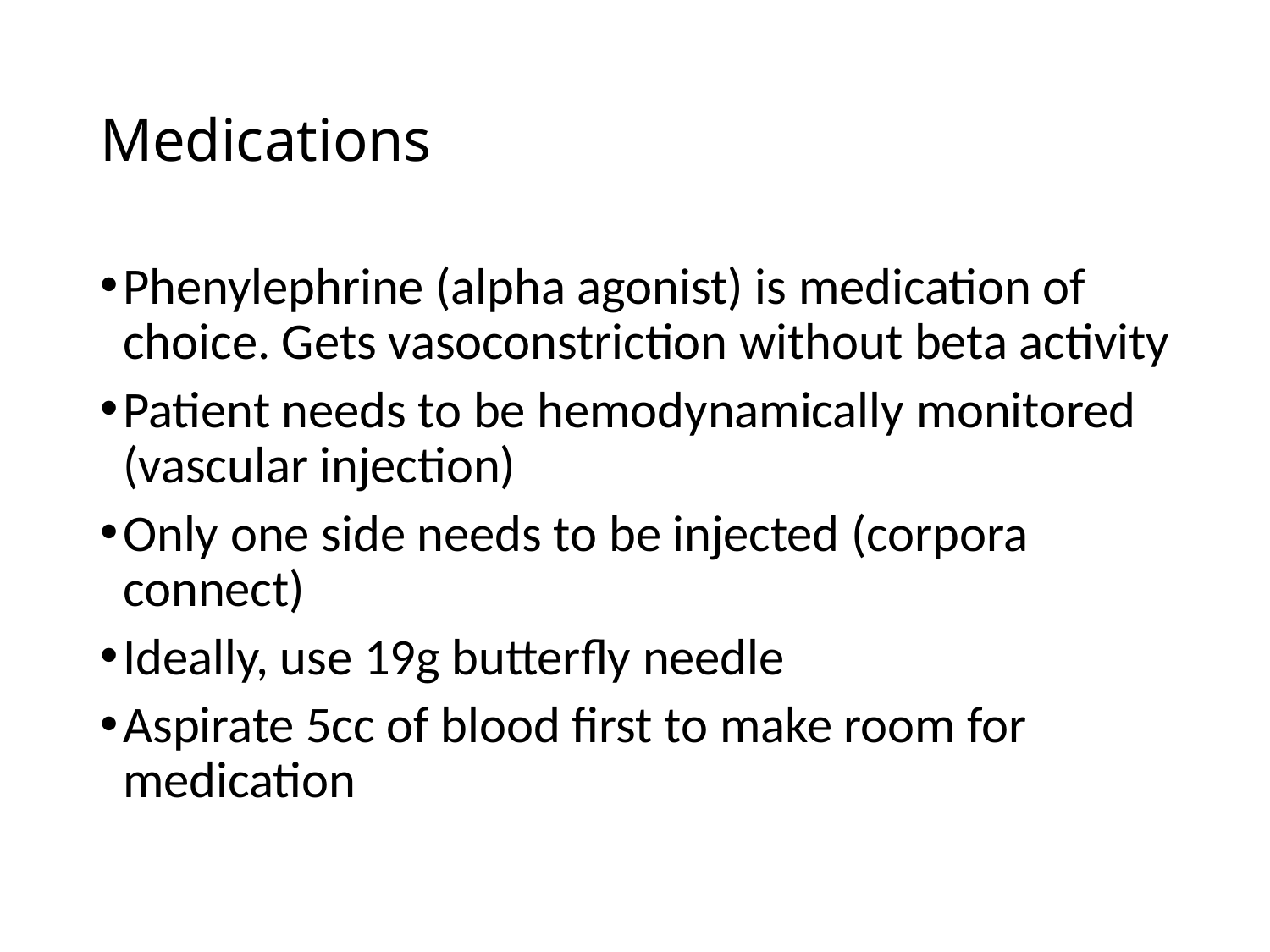

# Medications
Phenylephrine (alpha agonist) is medication of choice. Gets vasoconstriction without beta activity
Patient needs to be hemodynamically monitored (vascular injection)
Only one side needs to be injected (corpora connect)
Ideally, use 19g butterfly needle
Aspirate 5cc of blood first to make room for medication

## Slide 32
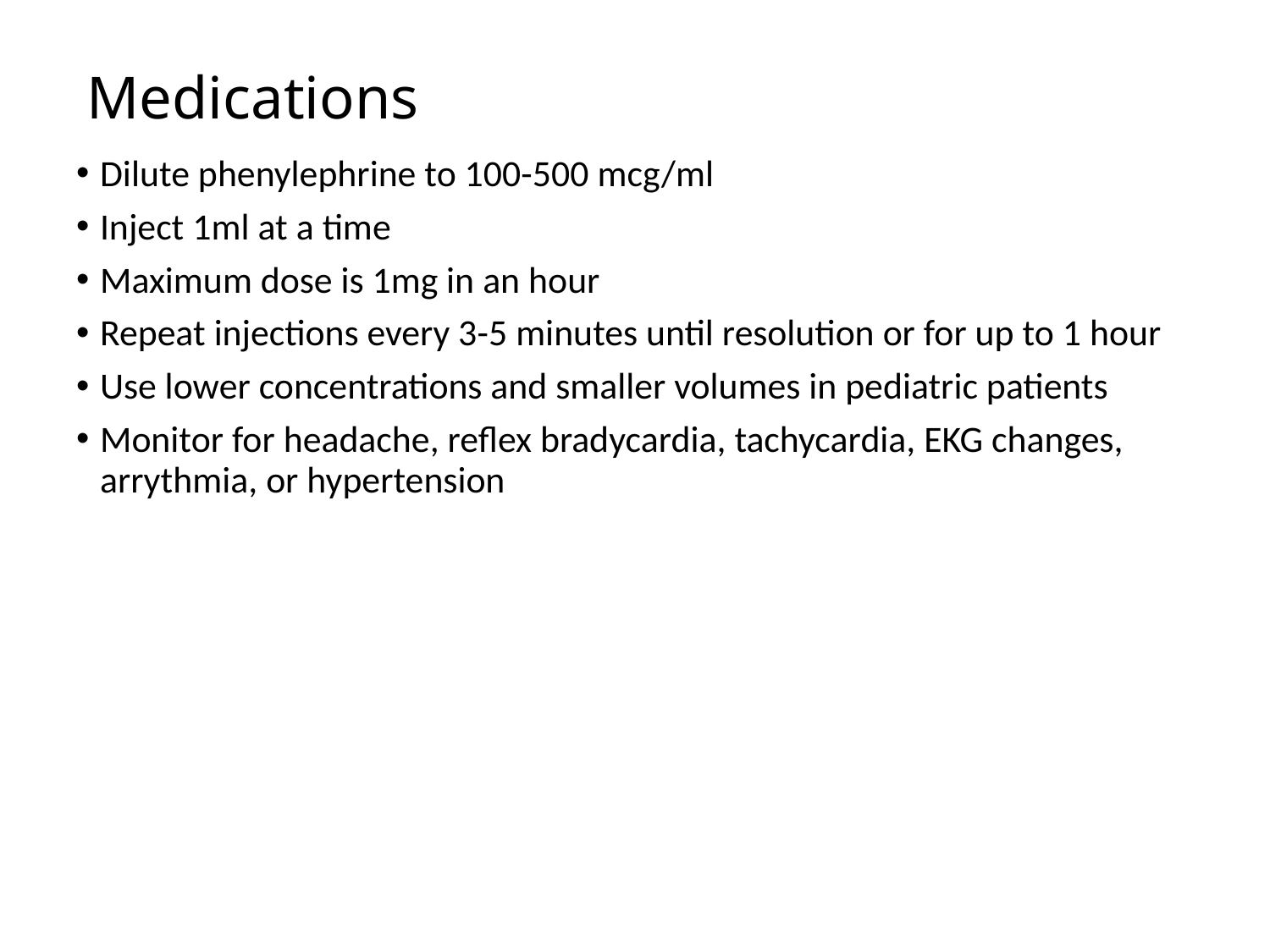

# Medications
Dilute phenylephrine to 100-500 mcg/ml
Inject 1ml at a time
Maximum dose is 1mg in an hour
Repeat injections every 3-5 minutes until resolution or for up to 1 hour
Use lower concentrations and smaller volumes in pediatric patients
Monitor for headache, reflex bradycardia, tachycardia, EKG changes, arrythmia, or hypertension

## Slide 33
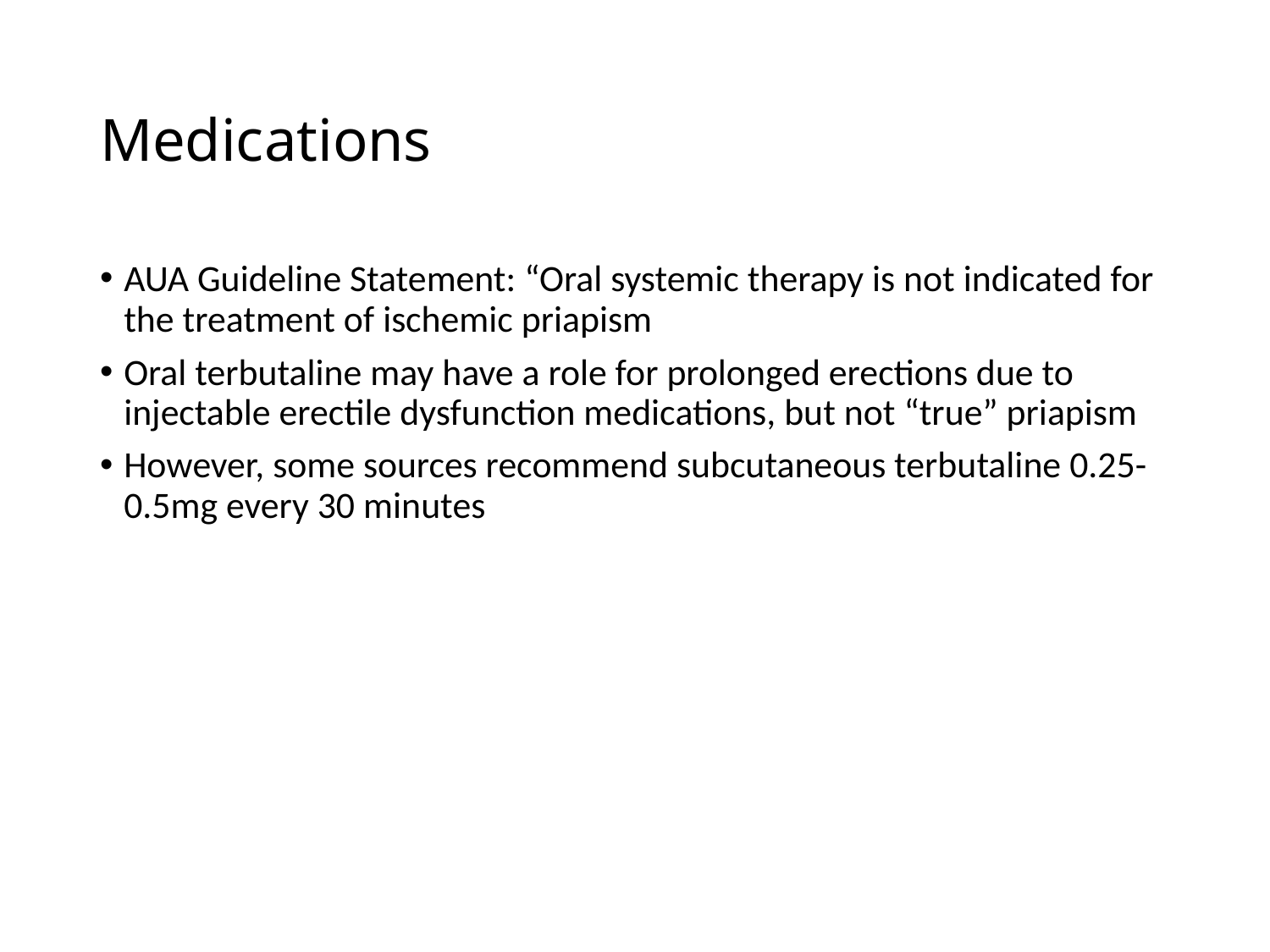

# Medications
AUA Guideline Statement: “Oral systemic therapy is not indicated for the treatment of ischemic priapism
Oral terbutaline may have a role for prolonged erections due to injectable erectile dysfunction medications, but not “true” priapism
However, some sources recommend subcutaneous terbutaline 0.25-0.5mg every 30 minutes

## Slide 34
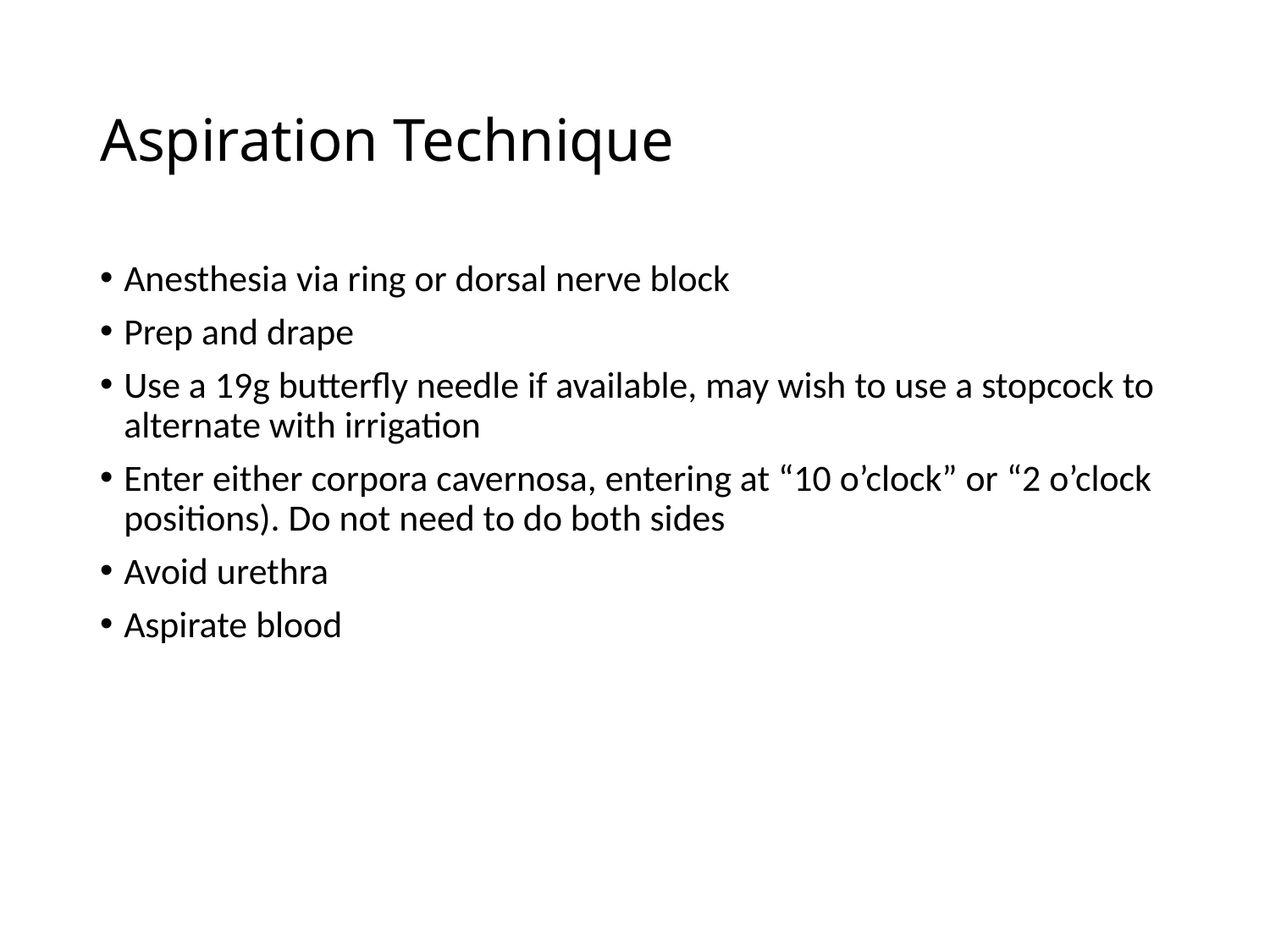

# Aspiration Technique
Anesthesia via ring or dorsal nerve block
Prep and drape
Use a 19g butterfly needle if available, may wish to use a stopcock to alternate with irrigation
Enter either corpora cavernosa, entering at “10 o’clock” or “2 o’clock positions). Do not need to do both sides
Avoid urethra
Aspirate blood

## Slide 35
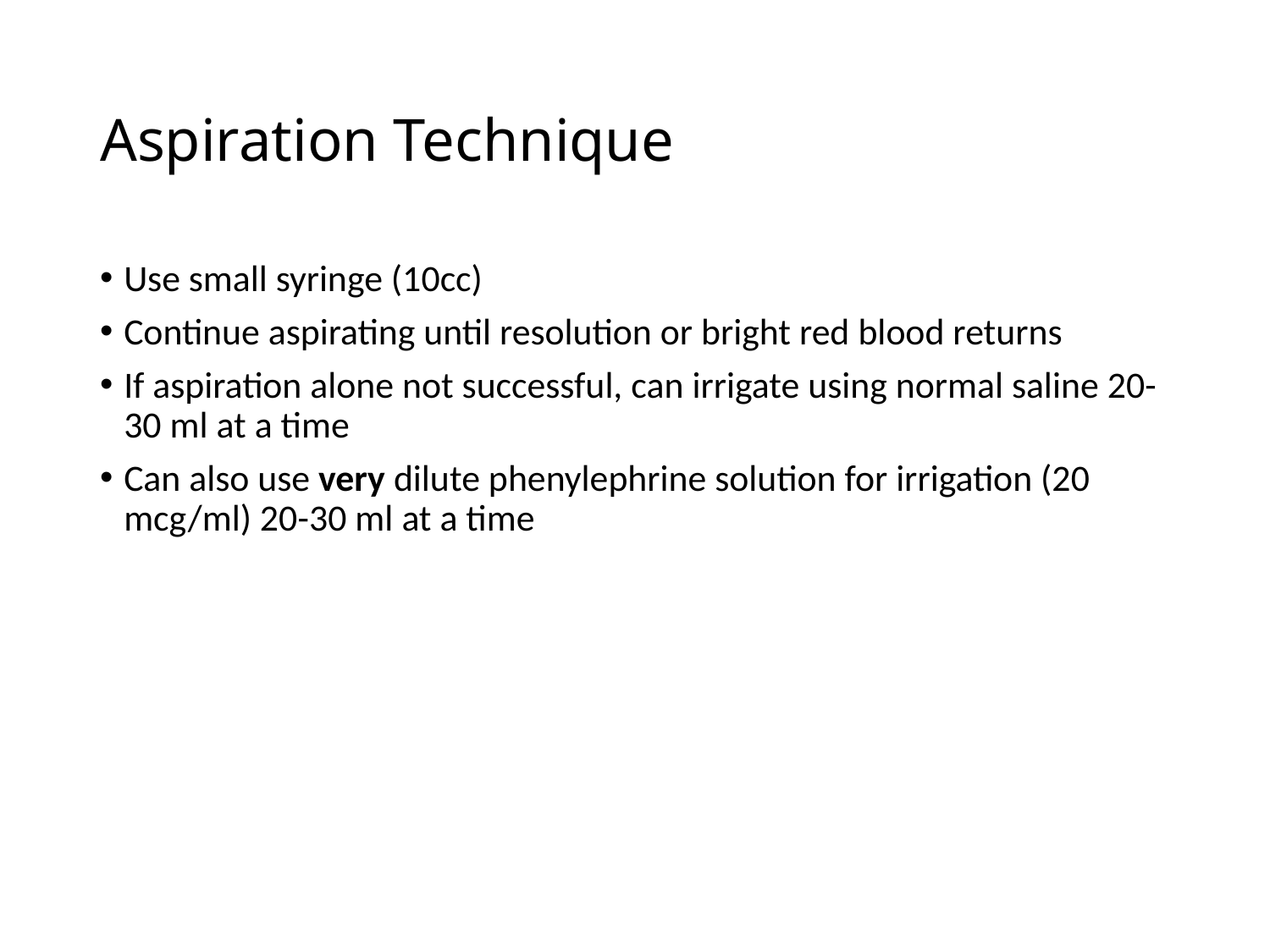

# Aspiration Technique
Use small syringe (10cc)
Continue aspirating until resolution or bright red blood returns
If aspiration alone not successful, can irrigate using normal saline 20-30 ml at a time
Can also use very dilute phenylephrine solution for irrigation (20 mcg/ml) 20-30 ml at a time

## Slide 36
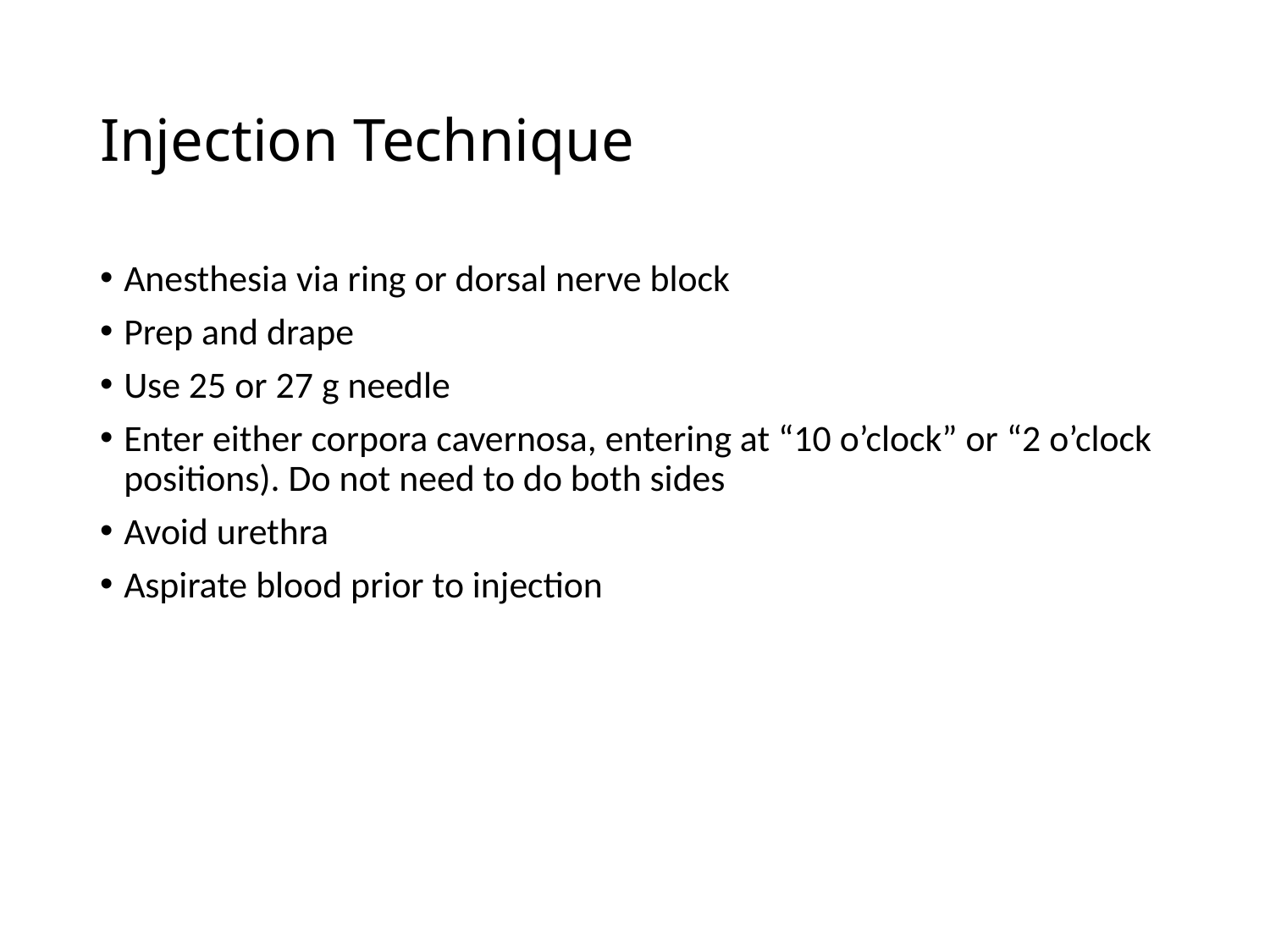

# Injection Technique
Anesthesia via ring or dorsal nerve block
Prep and drape
Use 25 or 27 g needle
Enter either corpora cavernosa, entering at “10 o’clock” or “2 o’clock positions). Do not need to do both sides
Avoid urethra
Aspirate blood prior to injection

## Slide 37
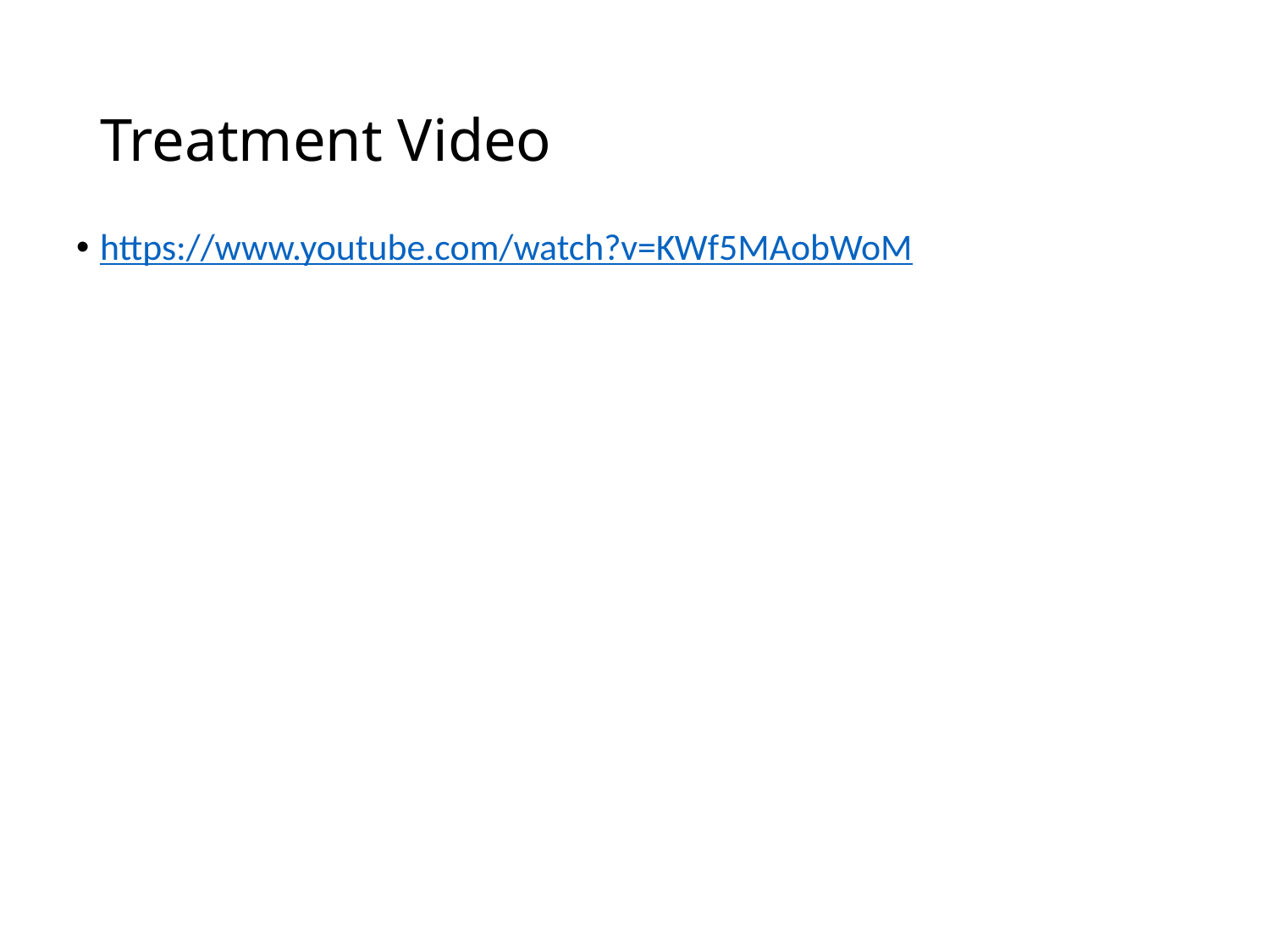

# Treatment Video
https://www.youtube.com/watch?v=KWf5MAobWoM

## Slide 38
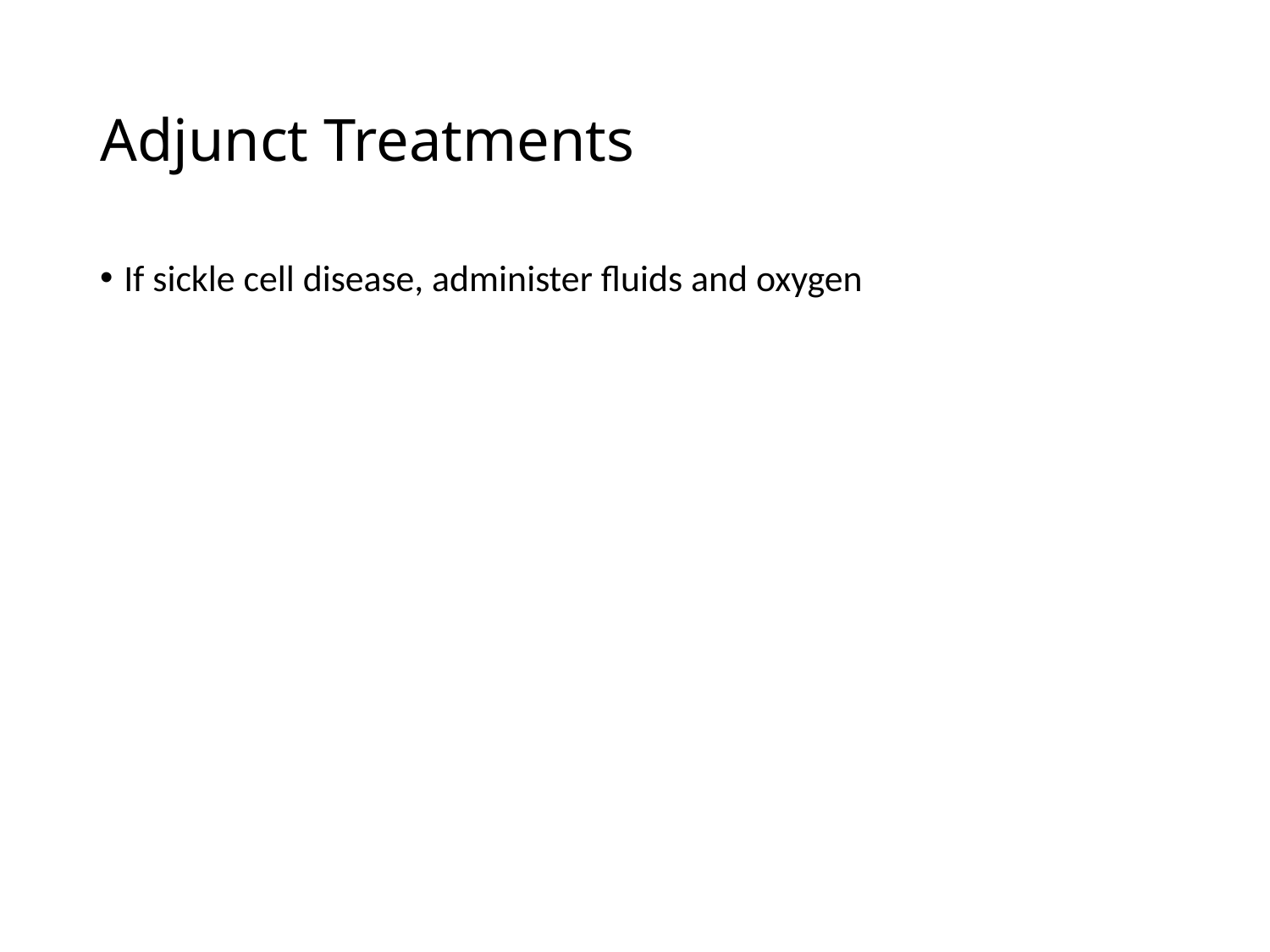

# Adjunct Treatments
If sickle cell disease, administer fluids and oxygen

## Slide 39
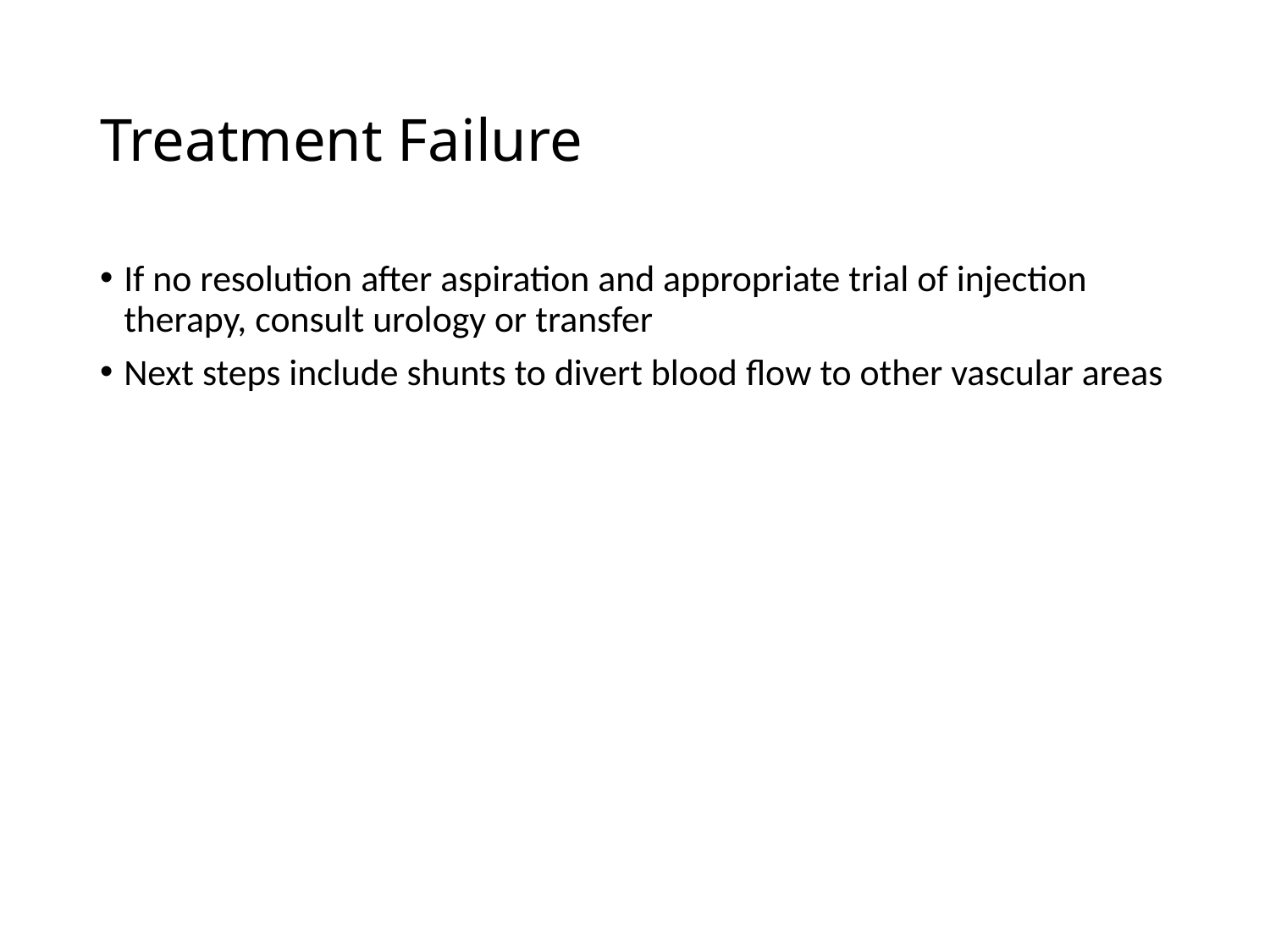

# Treatment Failure
If no resolution after aspiration and appropriate trial of injection therapy, consult urology or transfer
Next steps include shunts to divert blood flow to other vascular areas

## Slide 40
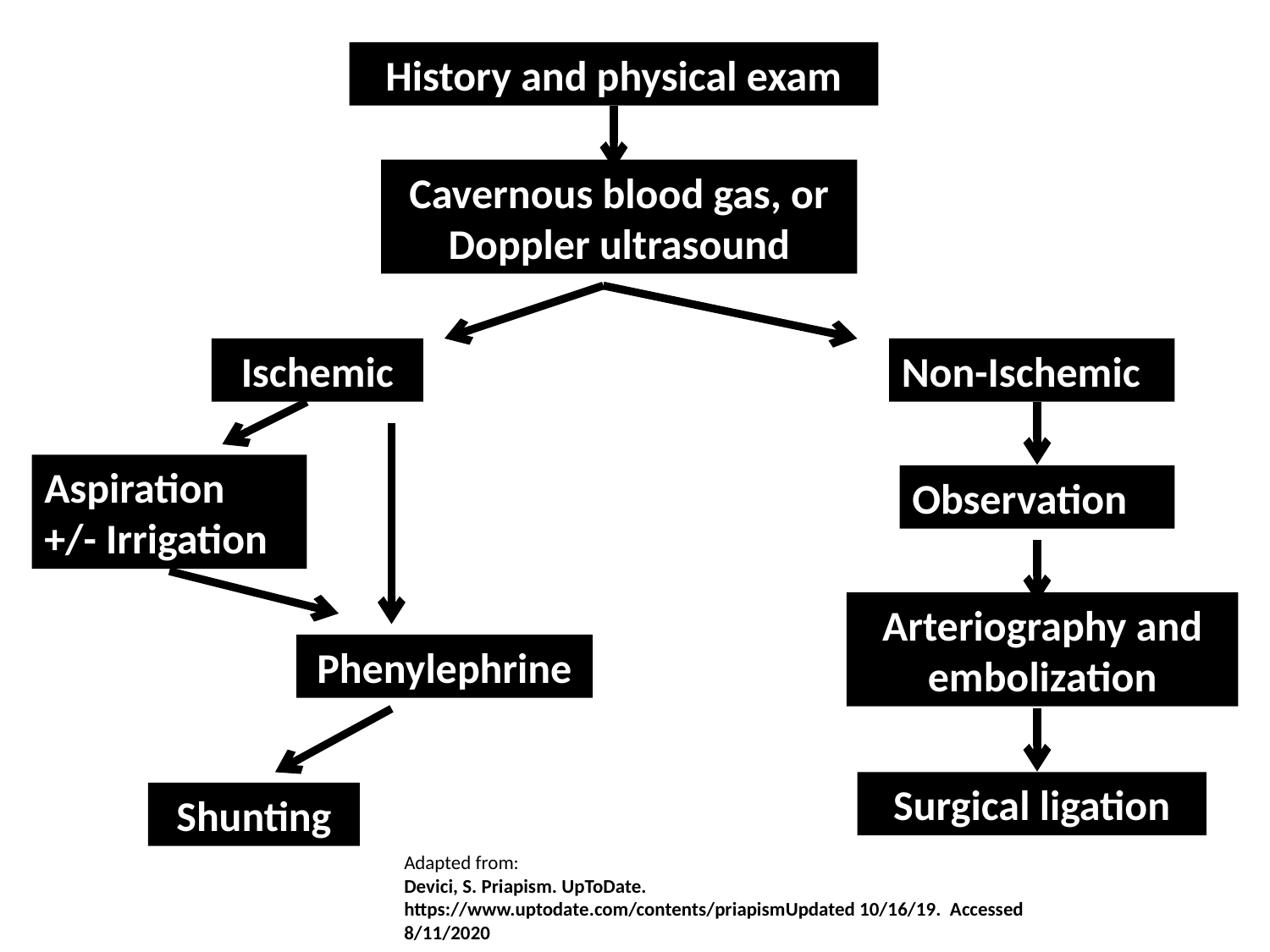

History and physical exam
Cavernous blood gas, or Doppler ultrasound
Ischemic
Non-Ischemic
Aspiration
+/- Irrigation
Observation
Arteriography and embolization
Phenylephrine
Surgical ligation
Shunting
Adapted from:
Devici, S. Priapism. UpToDate. https://www.uptodate.com/contents/priapismUpdated 10/16/19. Accessed 8/11/2020

## Slide 41
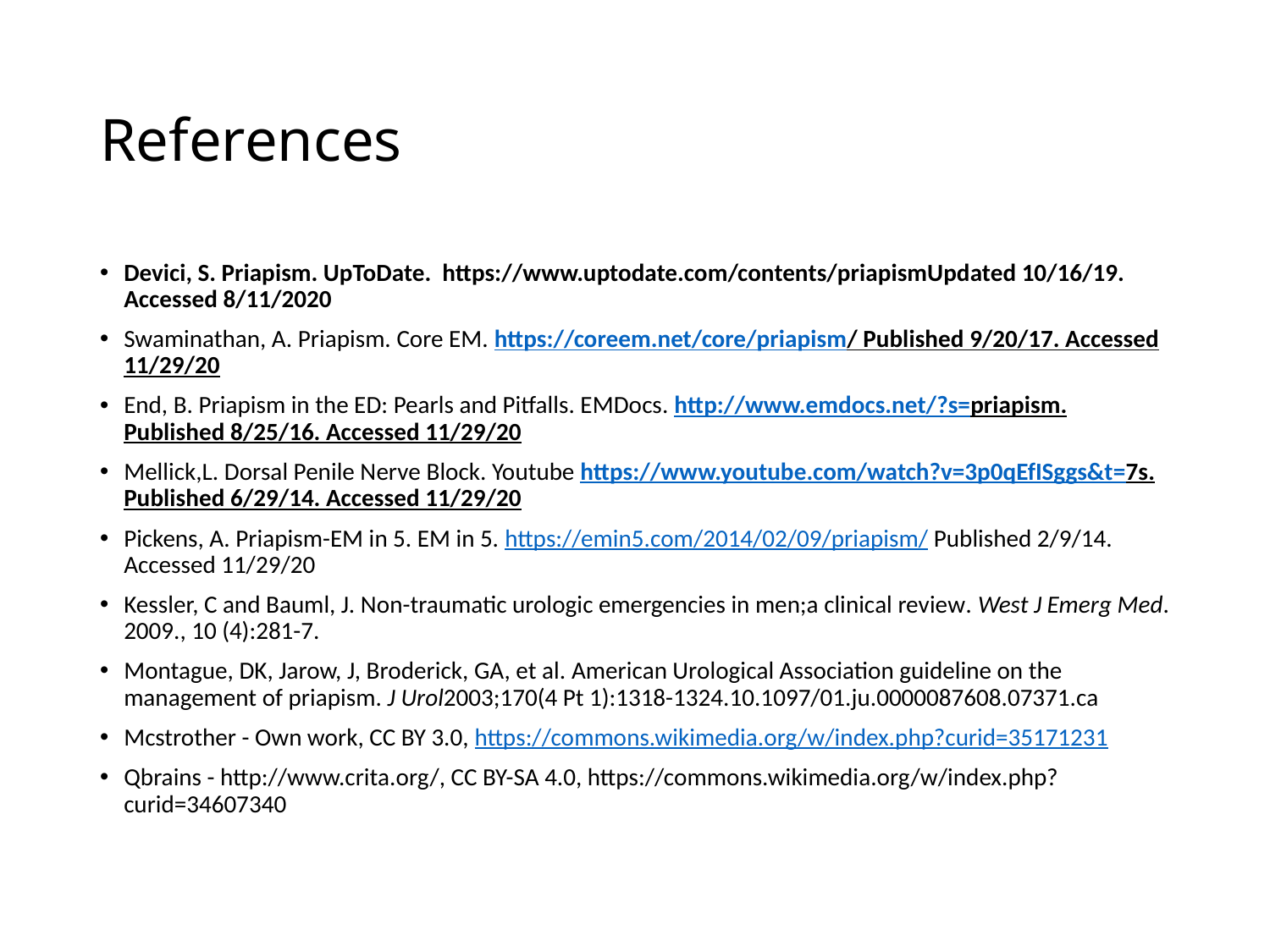

# References
Devici, S. Priapism. UpToDate. https://www.uptodate.com/contents/priapismUpdated 10/16/19. Accessed 8/11/2020
Swaminathan, A. Priapism. Core EM. https://coreem.net/core/priapism/ Published 9/20/17. Accessed 11/29/20
End, B. Priapism in the ED: Pearls and Pitfalls. EMDocs. http://www.emdocs.net/?s=priapism. Published 8/25/16. Accessed 11/29/20
Mellick,L. Dorsal Penile Nerve Block. Youtube https://www.youtube.com/watch?v=3p0qEfISggs&t=7s. Published 6/29/14. Accessed 11/29/20
Pickens, A. Priapism-EM in 5. EM in 5. https://emin5.com/2014/02/09/priapism/ Published 2/9/14. Accessed 11/29/20
Kessler, C and Bauml, J. Non-traumatic urologic emergencies in men;a clinical review. West J Emerg Med. 2009., 10 (4):281-7.
Montague, DK, Jarow, J, Broderick, GA, et al. American Urological Association guideline on the management of priapism. J Urol2003;170(4 Pt 1):1318-1324.10.1097/01.ju.0000087608.07371.ca
Mcstrother - Own work, CC BY 3.0, https://commons.wikimedia.org/w/index.php?curid=35171231
Qbrains - http://www.crita.org/, CC BY-SA 4.0, https://commons.wikimedia.org/w/index.php?curid=34607340
